# Barriers and Facilitators to Self-Care Behaviors in People Living with Osteoporosis: A Qualitative Descriptive Study

**DOI:** 10.3390/nursrep16010033

**Published:** 2026-01-20

**Authors:** Vicente Bernalte-Martí, Chiara Tedesco, Mara Tormen, Angela Cuoco, Gianluca Pucciarelli, Ercole Vellone, Maddalena De Maria, Emanuela Basilici Zannetti, Noemi Cittadini, Annalisa Pennini, Rosaria Alvaro

**Affiliations:** 1 Department of Nursing, Faculty of Health Sciences, Universitat Jaume I, 12071 Castellón, Spain; 2 Department of Biomedicine and Prevention, University of Rome Tor Vergata, 00133 Rome, Italy; chiara.tedesco.17@students.uniroma2.eu (C.T.); mara.tormen@students.uniroma2.eu (M.T.); gianluca.pucciarelli@uniroma2.it (G.P.); ercole.vellone@uniroma2.it (E.V.); emanuela.basilicizannetti@poliziadistato.it (E.B.Z.); noemi.cittadini@ptvonline.it (N.C.); annalisa.pennini@alumni.uniroma2.eu (A.P.); rosaria.alvaro@uniroma2.it (R.A.); 3 IRCCS Istituto Ortopedico Rizzoli, 40136 Bologna, Italy; angela.cuoco@alumni.uniroma2.eu; 4 Division of Research Methodology, Department of Nursing, Faculty of Nursing and Midwifery, Wroclaw Medical University, 50-996 Wroclaw, Poland; 5 Department of Life Science, Health, and Health Professions, Link Campus University, 00165 Rome, Italy; m.demaria@unilink.it

**Keywords:** barriers, facilitators, nursing, osteoporosis, qualitative interviews, self-care

## Abstract

**Background/Objectives**: Self-care is central to chronic illness management and is particularly relevant in osteoporosis to prevent complications and improve quality of life. Grounded in Riegel’s middle-range theory of self-care of chronic illness, the study sought to understand the contextual, emotional, and structural influences shaping self-care in people living with osteoporosis. **Aim**: The aim of this study was to explore patient-reported barriers and facilitators to self-care behaviors among individuals living with osteoporosis. **Methods**: A qualitative descriptive design was conducted using in-depth, semi-structured interviews with 20 patients with osteoporosis recruited via convenience sampling. Data were coded deductively and analyzed using Mayring’s qualitative content analysis with a deductive approach. **Results**: Participants identified several factors related to both barriers and facilitators of self-care behaviors. Four barrier sub-themes emerged: ineffective coping strategies, difficulties in osteoporosis management, inadequate physical activity, and ineffective self-efficacy. Six facilitator sub-themes were identified: self-care management strategies, osteoporosis management after a fracture, osteoporosis control, osteoporosis treatment, exercise, and confidence in one’s ability. Main barriers included fear of falling, ineffective self-efficacy, and poor care continuity, whereas key facilitators included support networks, motivation, and tailored care. **Conclusions**: Self-care behaviors in individuals with osteoporosis are influenced by emotional, contextual, and structural factors. Person-centered interventions integrating emotional and educational components may strengthen patients’ engagement and enhance self-care behaviors in osteoporosis. Identifying barriers and facilitators enables nurses to design empathetic, tailored strategies that enhance empowerment and disease management. Understanding these factors can improve autonomy for patients and adherence, promoting long-term health outcomes across clinical and community settings.

## 1. Introduction

Osteoporosis (OP) is a chronic metabolic bone disease characterised by the progressive loss of bone mineral density and the deterioration of bone microarchitecture, leading to increased bone fragility and a heightened risk of fractures, particularly among older adults and postmenopausal women [1,2]. The prevalence of OP in the world was reported to be 18.3% [3], with a notably high prevalence in women (23.1%). Fractures resulting from this condition constitute a major public health concern due to their association with increased morbidity and mortality, functional dependence, substantial socio-healthcare costs, and impact on quality of life [4,5].

Self-care plays a pivotal role in the management of chronic conditions such as OP, owing to its potential to prevent complications and enhance patients’ quality of life [6,7]. According to the Middle range Theory of Self-care of Chronic Illness [8,9], self-care is a naturalistic decision-making process that involves several behaviors that people with chronic diseases enact to maintain disease stability and to facilitate daily management. Despite well-documented benefits of self-care [10,11], many individuals with OP face considerable challenges in consistently maintaining self-care behaviours and adhering to recommended health practices, largely due to a range of personal, social, and economic barriers [12].

Traditionally, studies on OP have primarily focused on quantitative measures such as bone mineral density and fracture risk or identifying deficits related to treatment adherence, often overlooking a comprehensive and subjective exploration of the personal experiences that shape self-care behaviours in these patients [2,13]. However, there is a growing interest in investigating factors such as barriers and facilitators from a qualitative perspective, which enables the identification of elements that allow patients to sustain effective self-care over the long term. This approach broadens the understanding of the phenomenon and highlights the relevance of individual and context-specific perceptions of self-care [14].

Despite prior work identifying barriers such as a lack of specific knowledge about the disease, financial difficulties in accessing treatment, limited social support, and low personal motivation [15,16]; and facilitators including education, social support, exercise programs, and relationships with healthcare professionals [17,18], qualitative evidence remains limited in capturing how individuals experience diagnosis, adherence, and day-to-day self-care management in osteoporosis [19]. This is relevant because these lived experiences shape how self-care is understood and enacted in practice [20].

To address this gap, the Middle-Range Theory of Self-Care of Chronic Illness, proposed by Riegel and colleagues [9] and recently updated [8], conceptualizes self-care as a process comprising self-care maintenance, monitoring, and management that is well-suited for understanding and analyzing the complexities of self-care in OP. This framework supports a holistic, process-oriented examination of barriers and facilitators rather than focusing on single behaviors in isolation. This perspective has been applied across chronic conditions such as heart failure [11], chronic obstructive pulmonary disease [21], type I or type II Diabetes mellitus [22], ostomy [7] or hypertension [23], and is relevant to OP, where day-to-day decisions and perceived vulnerability may substantially shape self-care.

Consequently, OP research still lacks in-depth qualitative evidence that captures the subjective and processual nature of self-care [24]. Understanding lived experience is essential because beliefs, emotions, and contextual constraints influence whether recommendations are adopted and sustained [25]. Therefore, this study aimed to enhance the understanding of self-care in people living with OP by exploring their personal perceptions and lived experiences, with a specific focus on identifying perceived barriers and facilitators.

## 2. Materials and Methods

### 2.1. Study Design

We adopted a qualitative descriptive design [26] to answer the main questions of this study. To do so, this research design followed a structured, systematic methodological process [27]. First, we defined a concrete research question grounded in clinical practice, including explicit hypotheses and acknowledgment of researcher preconceptions. Second, the Middle-Range Theory of Self-Care of Chronic Illness [9] was used as a theoretical framework. This theory describes the concept of self-care as a naturalistic process of three main dimensions, and it informed (a) the development of the semi-structured interview guide, which was designed to elicit experiences related to self-care and its barriers/facilitators, and (b) the analytic strategy, through an a priori deductive coding framework based on Riegel’s self-care concepts and prior OP experience literature. Third, a convenience sampling strategy was employed to carefully select participants who provided rich, detailed descriptions of their lived experiences managing OP. Then, data processing and the presentation of results were conducted systematically, clearly addressing and reflecting upon the initial research question. The codebook was iteratively checked against the data and refined during team coding to ensure alignment with the study aim. Finally, we critically discussed the findings in relation to established quality criteria, ensuring transparency, credibility, and the applicability of our conclusions to clinical practice and theory advancement.

### 2.2. Participants and Setting

We used convenience recruitment from the outpatient clinic. Within this pool, we aimed for maximum variation across key demographic and clinical characteristics (e.g., age, education, employment status, caregiver, and osteoporosis type) to capture a range of experiences; however, variation was limited in some dimensions (e.g., gender) [28,29]. The inclusion criteria [14] were: (1) patients with a primary diagnosis of OP (either senile or postmenopausal); (2) aged ≥ 65 years for men and ≥50 years or postmenopausal for women; (3) T-score < −2.5 standard deviations (SD) on bone mineral density assessment, that measures bone strength and helps evaluate the risk of OP and fractures; (4) being able to communicate and conduct interview and (5) willingness to take part in the study. We excluded those patients younger than the specified age thresholds, had secondary forms of OP, exhibited a T-score > −2.5 SD, experienced cognitive impairment, faced language barriers, did not provide informed consent or withdrew from the study. The sample size was determined by data saturation, the point where no new themes were obtained during additional interviews [30,31]. Patients were recruited from the orthopedic outpatient clinic of the Tor Vergata Polyclinic, located in central Italy.

Of the 74 patients screened, 23 provided initial consent to participate. Among those who did not proceed to interview (n = 51), reasons included inability to use technological devices required for videocall interviews (as interviews were conducted via videocall), having osteopenia instead of osteoporosis, or lack of interest in research participation or in giving interviews. Of the 23 who initially consented, three later withdrew due to loss of interest and difficulties with personal organization. Therefore, 20 participants completed the semi-structured interviews in Italian. Data saturation was deemed to have been reached after 20 interviews because no new information was offered and no new themes were observed.

### 2.3. Data Collection

Sociodemographic and clinical data were collected via face-to-face self-administered questionnaires between March and June 2023. The first three interviews were used as a pilot test to determine whether the question pool was suitable for investigating the research question and whether it was well understood by the participants. Based on pilot feedback, the wording of the questions was simplified to improve clarity and comprehensibility. The semi-structured interviews were conducted via video calls (using the WhatsApp© VoIP System) and audio recorded. During the interviews, participants were all in their own homes and were invited to stay in a quiet and comfortable room. Two interview questions on facilitators and barriers were asked to each participant based on the Middle-Range Theory of Self-care by Riegel et al. [9]. The final interview guide is provided in Table 1. All Interviews were conducted by four researchers (CT, RN, PhD student, female) who were trained and had no relationship with the participants prior to the study’s commencement. Interviews lasted for 30 min on average. All interviews were audiotaped and transcribed, applying a pure verbatim protocol [27]. To ensure that recording was complete and accurate, one of the researchers produced and collected handwritten field notes during the interviews in paper note form. General impressions and specific features of the individual interviews were noted to generate additional information to inform the data analysis process. Transcriptions were not returned to the participants, although after the interview, a recap of what was discussed was provided for any comments or corrections.

### 2.4. Data Analysis

To analyze the textual data using qualitative content analysis, a structured seven-step deductive approach was applied, following the methodological framework proposed by Mayring [27]. The research questions and theoretical assumptions were clarified, focusing on self-care concepts according to Riegel et al. [9] and previously described experiences in OP literature. Subsequently, we defined a priori the category system and built a codebook composed of 72 codes grouped in 10 categories and having as main themes the concepts of self-care. Each code was related to an anchor sample and a definition extracted from Riegel’s self-care theory [9] and previous studies about the experience of osteoporotic disease. The coding guidelines were clearly defined, incorporating explicit definitions, anchor samples from textual data, and established coding rules based on Saldaña’s Emotion Coding method [32]. Four coders participated in this phase (CT, MT, VBM and AC), three of them with expertise in OP experiences (CT, MT and VBM) and one qualitative methods expert supervising the process, resolving discrepancies and ensuring consistency (AC). Initial interviews were coded, and preliminary codings were reviewed to verify if predefined codes adequately captured participants’ expressions. At the 50% mark of the material, the coders systematically revised the codebook and coding guidelines, adjusting as necessary to maintain alignment with the research objectives. Once all textual data (100%) were coded, the final coding was reviewed thoroughly to guarantee completeness and accuracy. In the final step, coders analyzed the frequency of each code and examined contingencies to determine which codes commonly appeared together in the participants’ narratives. Anchor examples, the final codebook, coding guidelines, and relevant methodological documents were compiled and made available as Supplementary Materials.

### 2.5. Study Rigour

The Lincoln and Guba criteria [33] were considered to ensure the study’s rigour: credibility, dependability, confirmability and transferability. Credibility of the research findings was ensured through extended and ongoing interaction with participants, facilitating a deeper and more comprehensive understanding of their lived experiences and perceptions of illness. Dependability was enhanced by providing a meticulous and explicit description of the data collection procedures and analytical methods, thus enabling transparency and the potential replication of the research. Confirmability was strengthened by involving a multidisciplinary research team composed of experts with diverse backgrounds and extensive experience in self-care practices related to chronic illness, which minimized potential researcher bias. Finally, a thorough and detailed description of the participants’ experiences and their cultural behavior was included to improve the transferability and applicability of the findings to other contexts [34].

Transcriptions were not returned to the participants; however, after each interview a recap of what was discussed was provided to allow comments or corrections. In addition, credibility and confirmability were supported through team-based coding and an explicit audit trail, including the final codebook and information about studies related to code creation (Supplementary S1), and anchor examples (Supplementary S2).

### 2.6. Ethical Considerations

The research received ethical approval from the Independent Ethics Committee of the Tor Vergata Polyclinic (registration number 211.22). All participants signed informed consent for study participation under the guidance of the researchers.

To improve the translation of patient quotations, ChatGPT-4o (OpenAI, San Francisco, CA, USA) was used. The approach assisted in ensuring accurate and contextually suitable translations since none of the writers are native English speakers, especially considering the source texts’ use of dialectal and colloquial terminology.

## 3. Results

### 3.1. Participants’ Demographics

Regarding the sociodemographic variables (Table 2), the study sample consisted of a total of 20 individuals, including 19 women and one man, aged between 55 and 78 years, all of Italian nationality.

Concerning marital status, the majority of the sample (65%, n = 13) were married or cohabiting. The most frequently reported education level was high school (45%, n = 9), followed by lower secondary school (40%, n = 8). The most common occupational status within the sample was retired, accounting for 60% (n = 12) of the cases.

Regarding the family context, most participants reported having a sufficient (55%, n = 11) or good (40%, n = 8) household income. Additionally, 65% (n = 13) of the sample lived with their spouse, who, in 45% (n = 9) of these cases, also served as their caregiver.

Finally, 80% (n = 16) of the participants reported having children, with an average of two per person in 60% (n = 12) of the cases.

### 3.2. Themes

In relation to the theme of Barriers, three sub-themes were derived from the content analysis, each containing different codes: (1) Ineffective coping strategies; (2) Difficulties in osteoporosis management; (3) Physical activity and self-efficacy. Moreover, in relation to the theme of Facilitators, six sub-themes were identified: (1) Self-care management strategies; (2) Osteoporosis management after a fracture; (3) Osteoporosis control; (4) Osteoporosis treatment; (5) Exercise; (6) Confidence in one’s ability. These two themes, along with their corresponding sub-themes and codes, are listed in Table 3 and allowed us to explore the OP experiences of the study participants.

### 3.3. Barriers

This theme summarizes barriers to OP self-care reported by participants. The sub-themes describe recurring difficulties identified across interviews and are illustrated with representative quotations.

#### 3.3.1. Ineffective Coping Strategies

Many patients adopt a passive attitude, relying almost exclusively on medical personnel and showing little initiative in managing their condition. This lack of coping skills is reflected in inconsistent behaviours, low motivation, and poor awareness of their health status. Emotionally, psychological distress is common, characterised by anxiety, anguish, and a pervasive sense of vulnerability. These feelings disrupt daily peace of mind and reduce the willingness to engage in proactive behaviours. Participants frequently described fear of falling and fear of fractures, which led them to restrict movement and feel insecure in daily activities.

*“I’m afraid that with one of my exaggerated movements I might, I might, I might break something, you see”*

(OP001, female, 66–70)

*“That’s mainly my problem, really, and then this creates anxiety, I’d say (sighs), I try to do it but then I get anxious, so I don’t know… now, for example, even when I have to give myself these injections, because I have to do them myself, but if I make a mistake, if I hurt something…”*

(OP010, female, 55–60)

*“This gives me a sense of insecurity and instability”*

(OP017, female, 55–60)

#### 3.3.2. Difficulties in OP Management

One of the most significant factors is the presence of adverse effects associated with treatment. This is compounded by a poor doctor–patient relationship, marked by insufficient communication, unclear consultations, and limited follow-up, leading to frustration and mistrust. Access to care is also hindered by long waiting times and frequent turnover of healthcare professionals, making treatment continuity difficult. Many patients fail to adhere properly to therapeutic recommendations due to memory problems, fear of side effects, or aversion to medication, even when they are aware of the need for treatment. Active disease prevention remains limited, and patients receive insufficient guidance on adopting protective behaviours. Added to this is considerable uncertainty regarding treatments, both in terms of their benefits and potential risks. The presence of comorbidities further complicates therapeutic management, interfering with dietary, pharmacological, and physical decisions.

*“But after a while, it was that injection you have to take every day. I started feeling cramps, I mean in my legs, and in fact, they stopped it for me”*

(OP008, female, 66–70)

*“Well, I didn’t know this, for example, because when they call you in for these appointments, they should also be much clearer; they should say: ‘Look, you’ll be taking this medicine, and this medicine might cause this. Be careful with these signs.’ But instead, they don’t tell you anything”*

(OP012, female, 76–80)

*“Surely the waiting times for diagnoses; so, diagnostic tests could definitely be, let’s say, more accessible”*

(OP017, female, 55–60)

*“Now, the doctors there wanted to give me hormones, but I was quite, how can I say… suspicious”*

(OP020, female, 66–70)

#### 3.3.3. Physical Activity and Self-Efficacy

Participants frequently described insufficient or unclear information about osteoporosis (e.g., symptoms, complications, and treatment options) and uncertainty about which behaviours were safe or beneficial. This lack of clarity, together with low perceived control, was described as reducing engagement in recommended behaviours. Participants also reported barriers to maintaining regular physical activity due to pain, fatigue, functional limitations, and fear of falling or injury, as well as uncertainty about how to start or sustain an appropriate routine.

*“Well, nothing special, I do a bit of light exercise, like taking the stairs, always trying to keep moving a little, going for walks”*

(OP002, male, 61–65)

*“I did things that were very, very, very gentle, exactly because I was aware that it could cause a fall or, in any case, something could have happened to me”*

(OP017, female, 55–60)

*“I don’t even know what the signs and symptoms of OP are”*

(OP001, female, 66–70)

*“Nothing, I don’t do anything, I just take these pills and that’s it, I don’t do anything else, no physiotherapy, nothing at all”*

(OP007, female, 71–75)

*“You don’t feel anything, you know? There’s no pain anywhere, I mean… I have some pain, but does osteoporosis cause pain? … I repeat, since I don’t feel any pain, it’s hard to stay on top of check-ups, you know? Because I don’t feel anything”*

(OP015, female, 61–65)

### 3.4. Facilitators

This theme summarizes facilitators of OP self-care reported by participants. The sub-themes capture resources and strategies described as helpful in day-to-day management and are supported by quotations.

#### 3.4.1. Self-Care Management Strategies

This sub-theme emerges as a key facilitator in the management of OP. Some participants identified faith as a strategy for dealing with pain, noting that spiritual reliance provided them with relief and a sense of calm. Others adopted a more pragmatic approach, choosing not to focus on the disease, which allowed them to coexist with it without falling into anxiety and to maintain emotional balance. Participants also emphasized the need for targeted information campaigns, access to regular screening tests, the intake of supplements such as vitamin D and calcium, and the adoption of an active lifestyle.

*“You shouldn’t become, you know, obsessive in that sense, I mean constantly thinking about your illness, whatever it may be, really”*

(OP005, female, 55–60)

*“I always say: make sure you go to the doctor, get something for your bones, because it’s not a nice thing, really. Yes, I tell all my friends: ‘make sure you get a bone scan, because… Yes, I say it”*

(OP010, female, 55–60)

*“But I don’t, thank God, I don’t suffer”*

(OP019, female, 71–75)

#### 3.4.2. OP Management After a Fracture

Following a fracture, affected patients highlighted the importance of attending regular medical check-ups, such as bone densitometry and blood tests, considering these actions essential for monitoring their health status. Several reported having taken the initiative to educate themselves and gain a better understanding of their condition, actively seeking information and following specific recommendations. Some participants also emphasised the value of the emotional support received after the fracture and underscored the role of the orthopedic specialist as a central figure in the recovery process.

*“I also do the treatments, of course. When I fell, they put a cast on me, then I wore a brace, then I did the therapies. Of course, of course”*

(OP012, female, 76–80)

*“The follow-up tests, the bone scan… I mean, I do the usual check-ups that my doctor prescribes… Many people don’t know and advise you not to… not to do it, really. But I know how to judge who it’s coming from”*

(OP013, female, 66–70)

#### 3.4.3. OP Control

Most participants considered continuity of medical care and access to well-trained professional teams, particularly in specialized settings, as essential. Those with peer support networks, whether through family members or others affected by the same condition, reported better emotional adaptation to the disease. Several participants stated that maintaining a trusting relationship and open communication with healthcare professionals facilitated treatment adherence. Personal motivation and intrinsic commitment were also identified as important driving forces, along with efforts to adapt positively to changes and actively seek clinical information about their condition.

*“Personal needs to feel self-fulfilled, and that’s the first foundation of being human: if you feel satisfied, if you feel fulfilled… then you’re also able to face many other situations around you”*

(OP014, female, 61–65)

*“Having a center that follows you, that takes you in, that really takes responsibility and care for you… these are definitely the things that help me. Having such dedicated healthcare staff is essential, it’s fundamental”*

(OP016, female, 61–65)

#### 3.4.4. OP Treatment

Several participants indicated that treatments were better accepted when perceived as effective and safe, particularly when integrated into a comprehensive approach that combined medication, proper nutrition, and physical exercise. Some emphasised the importance of being informed in advance about potential side effects, as this helped them manage their expectations more effectively. Additionally, they noted that treatment affordability supported long-term adherence and that the ability to self-administer medication gave them a greater sense of control over their therapy. Finally, the use of visual reminders or established routines enabled them to incorporate treatments consistently into their daily lives.

*“In the morning, when I have to take alendronate, I don’t set out the other medicines, so just not finding the pill boxes by touch on the nightstand already reminds me. On top of that, I place the box of alendronate and an empty glass by the sink in the bathroom, because the first thing I do is brush my teeth to remind myself not to have breakfast. Otherwise, I’d have breakfast and then skip the alendronate. So I have these little tricks to help my memory”*

(OP001, female, 66–70)

*“Taking these medicines, I’ve seen that, all in all, it helps”*

(OP003, female, 66–70)

*“It’s like a candy, you put it in your mouth, let it melt, or chew it a bit, and then drink a little water”*

(OP004, female, 55–60)

*“You know very well what happens with prolonged use of medications—not just to the stomach lining but also to the intestines. So this combination of stomach protectors and probiotics has really helped me manage the symptoms better”*

(OP016, female, 61–65)

#### 3.4.5. Exercise

Most participants stated that physical activity was easier to engage in when carried out in structured settings, such as senior centers with professional supervision. They also reported that exercise provided psychological well-being, increased energy, and improved mood. Those with programs tailored to their abilities and preferences, especially involving physiotherapists or encouragement from peers, reported higher adherence. Some even mentioned practices such as yoga or meditation as key elements for enhancing body awareness and reducing disease-related stress.

*“Yes, the physiotherapist. I went to the orthopedist, and the orthopedist actually recommended the physiotherapist more, for the tips on how to do the exercises”*

(OP004, female, 55–60)

*“I’m aware that, a little bit, instinctively, you protect yourself and close up; when I realize it, I try to straighten up—stomach in, chest out—and that’s it”*

(OP006, female, 66–70)

*“Physical activity, luckily, is something that really suits me, so out of all the things I have to be careful with, this is the one that bothers me the least”*

(OP017, female, 55–60)

*“At the senior center, well… it’s the usual stuff they do, a bit of everything, mostly working on the joints, with lots and lots of walking, and they tell you ‘do this, do that’… so many, really a lot… no day is the same as the next”*

(OP018, female, 75–80)

#### 3.4.6. Confidence in One’s Ability

Participants described how their confidence in managing the disease improved through concrete actions, such as following a diet rich in calcium and vitamin D. Simple habits, like regular sun exposure, also made them feel more proactive in their self-care. Likewise, those who had received nutritional counselling reported greater security when making dietary decisions, which enhanced their sense of control over the disease and reinforced their self-efficacy.

*“Honestly, the sun all my life (laughs), because it brings well-being—it’s good for the mind, so for the spirit, and for the bones. That’s important. Whenever I have a couple of minutes, even just on the balcony, I go out and sit in the sun. That’s something truly essential, absolutely indispensable”*

(OP014, female, 61–65)

*“Prefer foods that contain calcium and be very careful—really careful—with nutrition… Definitely eat healthy things, things that can help, help maintain calcium levels”*

(OP017, female, 55–60)

## 4. Discussion

The aim of this study was to explore facilitators and barriers of patients living with OP, using a qualitative descriptive approach grounded in Riegel’s middle-range theory of self-care of chronic illness [9]. To our knowledge, this is one of the few studies addressing patients’ experiences regarding their self-care practices, with a particular focus on the factors that hinder or support their implementation. Our findings add to the existing literature by providing novel insights into the lived experience of patients, highlighting underexplored psychosocial and contextual elements that shape self-care behaviors.

In our study, some of the predefined barriers and facilitators, constructed on a theoretical basis and derived from findings in other studies, did not emerge in our findings, which could be explained by the characteristics of the sample and the healthcare context. Since the participants were retired women with homogeneous sociocultural backgrounds and identical, universal access to the health system, the variability needed for certain barriers and facilitators such as ethnic differences, economic cost, or lack of time to emerge was limited. However, international studies show that these same factors do appear in more heterogeneous samples or in contexts with greater inequalities [35,36,37]. Thus, the “silence” of these codes in our study underscores the influence of context. Factors such as social support and trust in healthcare professionals may operate differently where healthcare access is fragmented, continuity is limited, or financial constraints are more salient; therefore, the prominence and meaning of these facilitators/barriers may differ in more socioeconomically constrained groups.

### 4.1. Barriers Faced by Individuals Diagnosed with OP

One of the initial findings of our research highlights the fear of falling when engaging in appropriate physical exercise, reflecting participants’ ongoing concern about bone fractures [38]. In a study involving women with OP and vertebral fractures, a combined exercise and education program reduced this fear, as evidenced by significant improvements in the Falls-Efficacy Scale-International (FES-I) at 3 and 12 months [39]. Participants reported that the persistent fear of falling is often accompanied by a perceived inadequacy regarding their physical abilities and a lack of specific knowledge about which activities are safe. This aligns with findings from other qualitative studies, where individuals stated, “I’m scared that I will fall during exercise. It hinders me to move” [40]. Ziebart et al. [37] and Shin et al. [41] emphasize that low exercise self-efficacy is a barrier to adopting physical activity recommendations. Moreover, reviews based on the Health Belief Model indicate that exercise self-efficacy predicts preventive behaviours such as physical activity [42].

There is also evidence of inefficient awareness regarding the importance of lifestyle modification, exacerbated by a superficial understanding of the disease and its implications [43]. Many participants reported becoming aware of their condition only at a later stage. Indeed, qualitative studies on postmenopausal OP show that women often perceive it as a slow process, “something normal with ageing”, and tend to underestimate its severity, which delays the initiation of active preventive measures [36]. Previous studies indicate that insufficient knowledge, coupled with limited communication with healthcare professionals, hinders engagement in protective behaviours such as regular exercise and appropriate nutrition [44]. Moreover, a lack of social support has been shown to intensify feelings of isolation and make it more difficult to sustain healthy behaviours, as noted in the literature [25]. The physiological process of ageing is perceived by patients as a barrier, leading them to normalize certain symptoms that may be related to OP, which negatively affects their ability to take health-promoting actions [45].

Regarding disease management, some participants expressed distrust toward medical advice due to poor communication and lack of follow-up. This, combined with potential comorbidities and insufficient knowledge, contributes to the development of misconceptions about the disease, ultimately hindering treatment adherence [46,47]. The existing literature confirms that the main factors affecting adherence include lack of knowledge [48,49], dissatisfaction with medical consultations [50], reduced confidence in the effectiveness of medication, and concerns about adverse effects [51,52,53]. A lack of clear information on symptoms and possible complications generates confusion and makes it harder for patients to adopt preventive behaviours [43]. A recent review highlights that strategies incorporating patient education, regular follow-up, and empowerment through shared decision-making improve adherence, in contrast to limiting beliefs that undermine it [54,55].

In terms of coping strategies, a tendency to delegate disease management to healthcare professionals was observed. The literature suggests that this passivity increases psychological vulnerability and anxiety, creating a vicious cycle that hinders active and calm management of the condition, and may lead to social isolation and the abandonment of preventive and management strategies for OP [56]. A clinical practice guideline noted that many patients “relied on healthcare staff rather than developing their own resources” and often restricted physical activity due to fear of falling, which further reinforced their emotional vulnerability [57]. A review on the psychological impact of OP documented that anxiety, fear of fractures, and social isolation are common and interrelated with avoidant behaviours, reinforcing a negative cycle [58]. Moreover, a recent study on patients with OP revealed considerable uncertainty about the disease, alongside a marked dependence on physicians for its management, which contributed to a passive attitude and lack of knowledge about self-care management [59].

### 4.2. Facilitators in OP Self-Care

The data from patients in our study highlighted awareness of the importance of a diet rich in calcium and vitamin D, reinforced through nutritional counselling, as a key facilitator for the optimal management of OP. The literature indicates that calcium and vitamin D remain essential pillars in reducing fracture risk [60], although many patients do not receive adequate guidance to meet the recommended daily intake or fully understand their clinical relevance [61]. Targeted nutritional counselling, along with strategies such as meal planning and online grocery shopping, has been shown to improve adherence to dietary recommendations for bone health [62].

Furthermore, analysis of the interviews revealed that participants only engaged in physical activity when it was adapted to their individual abilities and supported by tailored programs, the involvement of qualified professionals, and a socially stimulating environment. These factors were found to enhance motivation, self-esteem, and confidence, promoting more sustained and satisfying participation [4,63,64], while also reducing isolation and improving perceived safety [65].

In relation to the treatment of osteoporotic disease, our findings indicate the use of strategies such as reminders and self-motivation techniques by affected individuals to support adherence. These tools facilitate therapeutic continuity and the long-term maintenance of healthy habits [66,67]. The development and validation of instruments such as the Osteoporosis Self-Efficacy Scale [68] underscore the importance of self-confidence in initiating and sustaining preventive behaviours such as exercise and calcium intake, both crucial for long-term adherence. Moreover, higher self-efficacy has been significantly associated with better engagement in bone-protective behaviours and improved quality of life, indicating a clear link between personal confidence and sustained care [69].

Trust in healthcare professionals and a personalized approach have also emerged as key elements in disease management, enhancing therapy adherence and clinical outcomes, in line with the findings of Tarantino et al. [70] and Singer et al. [71]. Continuity of care, including regular monitoring through scheduled appointments or follow-up calls, has been reported as a facilitator for treatment stability and effectiveness, helping to prevent early discontinuation [72,73].

Our findings suggest that awareness of one’s own health status and self-information are fundamental for initiating and maintaining pharmacological treatments. In line with results from other studies conducted with older adults diagnosed with OP, awareness and access to information, reinforced by educational campaigns [49] and regular screening activities, promote preventive behaviours, greater responsibility, and self-care [35,74,75]. Finally, social support and a sense of belonging to support networks were reported as key facilitators; these factors help reduce isolation and improve psychological well-being, fostering a more positive attitude toward disease management and being associated with higher quality of life and mental health [76].

### 4.3. Strengths, Limitations and Future Research

This qualitative study provides a comprehensive and nuanced understanding of the barriers and facilitators influencing self-care among individuals with OP. One of its principal strengths lies in its focus on the subjective experience of the participants, which enabled the identification of emotional, relational, and contextual factors that are often overlooked in quantitative research. The systematic coding process, using a deductive approach, enriched the analysis and allowed for the capture of cultural and everyday aspects, such as care delegation, spirituality, and self-care management of treatment. Furthermore, the application of a robust theoretical framework offered interpretative coherence and facilitated the categorization of the findings around key dimensions such as self-efficacy, disease management, and physical activity.

However, the study also presents certain limitations. Although the sample size was sufficient to achieve theoretical saturation, it was relatively homogeneous in terms of cultural context and level of access to the healthcare system, which may restrict the transferability of the results to more diverse populations. Transferability may also be constrained by the recruitment and interview format: because interviews were conducted via videocall, individuals unable to use technological devices may be underrepresented. Moreover, the low proportion of screened patients who proceeded to interview suggests possible self-selection of participants who were more willing or able to engage in research participation, which could influence the salience of facilitators such as trust in healthcare professionals or engagement with follow-up. The Italian context of a tertiary outpatient clinic and universal healthcare access may attenuate barriers that are more prominent in socioeconomically constrained or resource-limited settings (e.g., access inequities), and the meaning of factors such as social support or trusting relationships may differ in more diverse populations. Additionally, data collection through individual interviews may have encouraged a rationalized narrative of self-care, potentially excluding more spontaneous or non-verbalized practices. Lastly, the absence of triangulation with other methods or stakeholders (such as caregivers or healthcare professionals) limits the relational depth of the analysis. Another limitation may be related to the predominantly female sample, which did not include adequate male representation. In addition, the questions were formulated in a general manner; however, having an all-female sample could have been leveraged as a strength to pose more specific, gender-oriented questions, such as how osteoporosis and menopause were managed.

Finally, there remains a need for further research that addresses patients’ subjective perceptions in order to design more effective interventions that are tailored to their realities and challenges.

## 5. Conclusions

In this predominantly older Italian sample recruited from a tertiary outpatient clinic, barriers to OP self-care were mainly related to fear of falling/fractures, limited disease knowledge and clarity of information, and discontinuity in follow-up, whereas key facilitators included supportive networks, trusting relationships with healthcare professionals, tailored education/exercise programs, and strategies that helped integrate treatment and lifestyle recommendations into daily routines. These findings support person-centered interventions that combine emotional support with structured, accessible education and continuity of care. At a service and public-health level, the results highlight the potential value of reinforcing educational campaigns and screening pathways and strengthening continuity across care interfaces to sustain long-term self-care.

## Figures and Tables

**Table 1:** Interview guide.

Self-Care in Elderly Patients with Osteoporosis: Descriptive Qualitative Study		
Factors Explored	Main Questions	Supporting Questions
Barriers and facilitators of self-care	What factors make it easier for you to manage your osteoporosis?	What difficulties did you encounter when changing your lifestyle after being diagnosed with osteoporosis?
	What factors prevent you from taking care of your osteoporosis?	What would you recommend to someone with osteoporosis to better manage their condition?
Free open question	Would you like to add anything else before the end of the interview?	

**Table 2:** Socio-demographic characteristics of the sample.

		Total Sample (n = 20)
Variables		Mean (SD)
Age (years)		67.25 (6.21)
		n (%)
Gender	Female	19 (95.00)
	Male	1 (5.00)
Marital status	Married	13 (65.00)
	Single	4 (20.00)
	Divorced	1 (5.00)
	Widowed	2 (10.00)
Education level	Elementary school	2 (10.00)
	Lower secondary school	8 (40.00)
	High school	9 (45.00)
	University degree	1 (5.00)
Employment status	Retired	12 (60.00)
	Employed	3 (15.00)
	Unemployed	3 (15.00)
	Housewife	2 (10.00)
Household Income	Good	8 (40.00)
	Sufficient	11 (55.00)
	Insufficient	1 (5.00)
Primary Caregiver	Spouse	9 (45.00)
	Daughter/Son	2 (10.00)
	Other	1 (5.00)
	None	8 (40.00)
Person with whom they live	Spouse	13 (65.00)
	Daughter/Son	2 (10.00)
	Alone	3 (15.00)
	Other	2 (10.00)
Children	1	3 (15.00)
	2	12 (60.00)
	3	1 (5.00)
	No	4 (20.00)

Legend. SD: Standard Deviation.

**Table 3:** Summary of the themes, sub-themes and codes categorized from the data.

Themes	Sub-Themes	Codes	Frequency
Barriers	Ineffective coping strategies	Lack of Coping skills	8
		Psychological Distress	6
		Fear of falling	9
		Fear of fractures	2
	Difficulties in osteoporosis management	Side effects	29
		Bad Relationship with healthcare providers	45
		Racial or ethnic differences	-
		Conflicting advice	10
		Disease-related symptoms	33
		Difficulty accessing care	46
		Non-adherence to therapy	15
		Gaps in prevention	14
		Uncertainty	37
		Impact in daily routine	18
		Comorbidities	16
		Osteoporosis being overlooked by other specialists	8
		Disagreement with osteoporosis treatment	3
		Financial constraints	2
		Cost of illness	12
	Physical activity and self-efficacy	Lack of disease prevention services	11
		Inefficient awareness	3
		Weak psychological support systems	-
		Cost of illness	-
		Personal capability	1
		Lack of exercise-related Knowledge	1
		Low exercise self-efficacy	4
		Bad rehabilitation professionals’ ability	-
		Lacking trust in rehabilitation workers	1
		Lack of time	-
		Lack of transportation	1
		Uncertainty	2
		Fear of falling	5
		Knowledge gaps	42
		Disease self-perception	31
		Disease social perception	6
		Aging	6
		Not exercising regularly	24
		Inadequate consumption of milk and dairy products	22
		Inadequate exposure to sunlight	2
		Being a caregiver for others	1
		Lack of support people/caregivers	8
		Inadequate unhealthy diet	2
		Inefficient awareness	44
		Self-neglect	23
		Personal capability	5
Facilitators	Self-care management strategies	Praying to relief pain	-
		Religious practices to relief pain	-
		Faith healing to relief pain	1
		Herbal remedies to relief pain	-
		Osteoporosis prevention	19
		Trying not to think about illness	3
	Osteoporosis management after a fracture	Testing after a fracture	21
		Informing after a fracture	5
		Tailored education after a fracture	1
		Support after a fracture	1
		Orthopedic advice after a fracture	12
		Racial or ethnic differences	-
		Continuity	40
	Osteoporosis control	Mutual help	11
		Peer support	20
		Good Relationship with healthcare providers	37
		Effective remedies	51
		Adaptation, Positive	85
		Networking for seeking information	11
		Clinical guidance	38
		Adequate support systems	1
	Osteoporosis Treatment	Safety	9
		Low out-of-pocket costs	2
		Self-administer	21
		Strategies to facilitate adherence	13
		Volition	26
	Exercise	Adequate network resources	3
		Positive emotions regarding physical activity	23
		Positive reactions regarding physical activity	23
		Customized exercises	27
		Encouragement in physical activity	11
		Guidance from physical therapists	8
		Mindful exercise	11
		Adequate consumption of milk and dairy products	7
	Confidence in one’s ability	Adequate exposure to sunlight	8
		Nutritional counseling	11

## Data Availability

Dataset is available upon request from the authors. The data are not publicly available due to [privacy and ethical restrictions].

## Supplementary Materials

The following supporting information can be downloaded at: https://www.mdpi.com/article/10.3390/nursrep16010033/s1, Supplementary S1: Codebook; Supplementary S2: Anchor samples [77,78,79,80,81,82,83,84,85,86,87,88,89,90,91,92].

## Public Involvement Statement

Patients were involved as study participants in this qualitative study; however, they were not involved in the design, conduct, analysis, or reporting of the research.

## Guidelines and Standards Statement

This manuscript was drafted against the COREQ checklist (Consolidated Criteria for Reporting Qualitative Research).

## Abbreviations

The following abbreviations are used in this manuscript:

OP	Osteoporosis
FES-I	Falls-Efficacy Scale-International
