# Supplementary material for: Barriers and Facilitators to Self-Care Behaviors in People Living with Osteoporosis: A Qualitative Descriptive Study
Source: Nurs Rep. 2026 Jan 20;16(1):33. doi: 10.3390/nursrep16010033 (PMC12844863; doi:10.3390/nursrep16010033)
Supplement: Supplementary file 1 [file nursrep-16-00033-s001.zip › Supplementary S1.pdf]

Supplementary S1. Codebook

THEME: BARRIERS		
**Code**	**Definition**	**Authors**
**Category: inadequate physical activity**		
Inefficient awareness	The act of "taking account" of an object or situation	Lyu et al., 2024
Personal capability	The ability or qualities necessary to do something	Ziebart et al., 2022
Lack of exercise-related knowledge	The information, understanding and skills that you gain through education or experience	Ziebart et al., 2022
Low exercise self-efficacy	Cognitive mechanism based on expectations or beliefs about one's ability to perform actions necessary to produce a given effect. It is also a theoretical component of behavior change in various therapeutic treatments	Ziebart et al., 2022
Lacking trust in rehabilitation workers	Mistrust somebody/something to have no confidence in somebody/something because you think they may be harmful; to not trust somebody/something	McArthur et al., 2018
Lack of transportation	Lack of public transportation to reach rehabilitation centers or facilities set up for physical activity	Rodrigues et al., 2017
Uncertainty	Being uncertainty about One's abilities and possibilities in performing the physical activity	Dohrn et al., 2016
Fear of falling	Is a sense of unease, is to be vulnerable, and is a call for help	Hamed et al., 2021
**Category: ineffective self-efficacy**		
Knowledge gaps	The body of truths or facts accumulated in the course of time, the cumulated sum of information, its volume and nature, in any civilization, period, or country	Gül et al., 2023
Disease self-perception	The process by which the nature and meaning of sensory stimuli are recognized and interpreted	Gül et al., 2023
Disease social perception	The perceiving of attributes, characteristics, and behaviors of one's associates or social groups	Gül et al., 2023
Aging	The gradual irreversible changes in structure and function of an organism that occur as a result of the passage of time	Gül et al., 2023
Not exercising regularly	Physical activity which is usually regular and done with the intention of improving or maintaining PHYSICAL FITNESS or HEALTH	Gül et al., 2023
Inadequate consumption of milk and dairy products	Raw and processed or manufactured milk and milk-derived products. These are usually from cows (bovine) but are also from goats, sheep, reindeer, and water buffalo	Gül et al., 2023
Inadequate exposure to sunlight	Irradiation directly from the sun	Gül et al., 2023
Being a caregiver for others	People who provide care to those who need supervision or assistance in illness or disability. They may provide the care in the home, in a hospital, or in an institution. Although caregivers include trained medical, nursing, and other health personnel, the concept also refers to parents, spouses, or other family members, friends, members of the clergy, teachers, social workers, fellow patients	MeSH
Lack of support people/caregivers	Positive influence of caregivers on bone health and on environmental safety	Zubick et al., 2024

Inadequate unhealthy diet	Do not feed on dietary patterns which have been found to be important in reducing disease risk	MeSH
Inefficient awareness	The act of taking account of an object or situation. It does not imply assessment of, nor attention to the qualities or nature of the object	MeSH
Self-neglect	Profound inattention by individuals to their own health and hygiene	MeSH
Personal capability	The ability or qualities necessary to do something	Ziebart et al., 2022
**Category: difficulties in osteoporosis management**		
Side effects	Stomach problems to violent nausea, vomiting bile, and burning […] Ways to reduce side effects would be likely to positively influence people's decisions to remain adherent to these medications	Salter et al., 2014
Bad Relationship with healthcare providers	The interactions between physicians and patient	Brod et al., 2008
Conflicting advice	Discordant advice from physicians about performing physical activity	Dohrn et al., 2016
Disease-related symptoms	Clinical manifestations that can be either objective when observed by a physician, or subjective when perceived by the patient	Ziebart et al., 2022<br>MeSH
Difficulty accessing care	Challenges in attending face-to-face appointments due to transportation, cost, and other barriers	Saag et al., 2021
Non-adherence to therapy	Non-compliance (taking medication inappropriately) with fracture prevention medication	Schousboe et al., 2013
Gaps in prevention	Ambiguity about osteoporosis prevention and reluctance to take anti-osteoporosis medication (AOM)	Barcenilla et al., 2020
Uncertainty	The condition in which reasonable knowledge regarding risks, benefits, or the future is not available	MeSH
Impact in daily routine	Difficulties in carrying out daily activities due to pain or change in the routines to cope with health problems	Al Zadjali et al., 2024
Comorbidities	The presence of co-existing or additional diseases with reference to an initial diagnosis or with reference to the index condition that is the subject of study. Comorbidity may affect the ability of affected individuals to function and their survival; it may be used as a prognostic indicator for length of hospital stay, cost factors, and outcome or survival	MeSH
Osteoporosis being overlooked by other specialists	Health professionals who do not deal directly with OP tend to consider it a benign disease, of low priority, whose management is not linked to their responsibility	Rezae et al., 2024
Disagreement with osteoporosis treatment	Concordance with osteoporosis treatment has been linked to a reduction in fracture risk. Despite this strategy's effectiveness, the literature shows that concordance with osteoporosis treatment is a common challenge faced by postmenopausal women worldwide	Al Zadjali et al., 2024
Financial constraints	Limitations on the availability of funds or financial resources that restrict an individual's ability to spend or invest	Muhamad Jamil et al., 2024
Cost of illness	The personal cost of disease which may be economic, social, or psychological. The cost of illness may be reflected in absenteeism, productivity, response to treatment, peace of mind, or quality of life	MeSH

Lack of disease prevention services	Deficiencies or shortcomings in the processes, strategies, or resources aimed at preventing diseases	Muhamad Jamil et al., 2024
**Category: ineffective coping strategies**		
Lack of Coping skills	Various techniques for actively managing a stressful event or situation	MeSH
Psychological Distress	Negative emotional state characterized by physical and/or emotional discomfort, pain, or anguish	MeSH
Fear of falling	A sense of anxiety regarding a falling incident	Delbari et al., 2023
Fear of fractures	Deep concern about bones fractures	Barker et al., 2016<br>Rothmann et al., 2018
**THEME: FACILITATORS**		
**Code**	**Definition**	**Authors**
**Category: Osteoporosis management after a fracture**		
Testing after a fracture	Instrumental, diagnostic or blood tests (e.g., BDM)	Majumdar et al., 2014
Informing after a fracture	Inquire about the management of fracture in osteoporosis after it has happened	Majumdar et al., 2014
Tailored education after a fracture	Receive personalized educational interventions	Feldstein et al., 2008
Support after a fracture	Organized efforts to address ongoing psychological and social problems of individuals, their partners, families and caregivers	Feldstein et al., 2008
Orthopedic advice after a fracture	Participation of orthopedic specialists in process of healing after a fracture	Feldstein et al., 2008
**Category: Osteoporosis control**		
Continuity	In healthcare encounters	Wright et al., 2019
Mutual help	Support founded in the women lived experiences	Nilsson et al., 2019
Peer support	Support founded in the women lived experiences	Nilsson et al., 2019
Good Relationship with healthcare providers	The interactions between physician and patient	Brod et al., 2008
Volition	Voluntary activity without external compulsion.	MESH
Adaptation, Positive	A state of harmony between internal needs and external demands and the processes used in achieving this condition	MESH
Networking for seeking information	In the category of establishing networking for seeking information, women with osteoporosis introduced television, Google search engine, Telegram, books, magazines, brochures, and posters as sources of information for their health	Ansari et al., 2021

Clinical guidance	The relevant literature shows that, during follow-up, tailored educational intervention along with counseling, motivational interviews, and educational programs are more effective than standard information at improving the outcomes of osteoporotic patients	Tarantino et al., 2017
**Category: Osteoporosis treatment**		
Effective remedies	Empirical evaluation of the effectiveness of certain remedies or medicines or activities	Beaudart et al, 2022
Safety	participants ranked the safety attributes in their top 1, top 2 and top 3 of most important attributes, respectively. Most patients understood the risk of side effects with osteoporosis treatments and expressed the importance of knowing what to expect before treatment initiation	Beaudart et al, 2022
Low out-of-pocket costs	Cheap remedies	Beaudart et al, 2022
Self-administer	Activities or medicines that can be delivered at home without the patient having to go to a health center	Beaudart et al, 2022
Strategies to facilitate adherence	Once patients accepted the need for treatment, they devised many of their own strategies for incorporating taking medications into their daily routines	Lau et al., 2008
**Category: Exercise**		
Adequate support systems	Organized efforts to address ongoing psychological and social problems of individuals, their partners, families and caregivers	Lyu et al., 2024
Adequate network resources	Organized efforts to address ongoing psychological and social problems of individuals, their partners, families and caregivers	Lyu et al., 2024
Positive emotions regarding physical activity	Those affective states which can be experienced and have arousing and motivational properties. Patients responded positively to exercise if it facilitates recovery from illness. As long as it has a positive effect on the rehabilitation of the disease, they were willing to try whether it is pharmacological or nonpharmacological therapy	Lyu et al., 2024
Positive reactions regarding physical activity	What do you do, say or think because of something that has happened. Patients responded positively to exercise if it facilitates recovery from illness. If it has a positive effect on the rehabilitation of the disease, they were willing to try whether it is pharmacological or nonpharmacological therapy	Lyu et al., 2024
Customized exercises	Exercise programs that meet needs and preferences	Ziebart et al., 2022
Encouragement in physical activity	Being encouraged by physical therapists, caregivers, friends, etc.	Dohrn et al., 2016
Guidance from physical therapists	Being guided in PA by physical therapists	Dohrn et al., 2016
Mindful exercise	Practice about mindfulness exercises, including mindful eating, body scan and meditation	Lyu et al., 2024
**Category: Confidence in one's ability**		
Adequate consumption of milk and dairy products	Raw and processed or manufactured milk and milk-derived products. These are usually from cows (bovine) but are also from goats, sheep, reindeer, and water buffalo	Gül et al., 2023
Adequate exposure to sunlight	Irradiation directly from the sun	Gül et al., 2023

Nutritional counseling	Adjust one's diet after undergoing a dietary or nutritional examination	French et al., 2008
**Category: Self-management strategies**		
Faith healing to relieve pain	The use of faith and spirit to cure disease	Zadjali et al., 2023
Osteoporosis prevention	Health communication campaigns are a type of media campaign that seeks to promote public health by developing educational health interventions. The purpose of such campaigns is to increase the individual’s awareness about the impacts of diseases and to provide them with more information regarding prevention methods	Dastmanesh et al., 2023
Trying not to think about illness	During the interview, participants commonly reported that having osteoporosis did not have much impact on them and they did not think about it daily	Besser et al., 2012

References:

Al Zadjali, F., Brooks, J., O'Neill, T. W., & Stanmore, E. (2024). Experiences of postmenopausal osteoporosis: a narrative review. *Disability and rehabilitation*, *46*(5), 828–840. https://doi.org/10.1080/09638288.2023.2169770

Ansari, A., Fahimfar, N., Noruzi, A., Fahimifar, S., Hajivalizadeh, F., Ostovar, A., Larijani, B., & Sanjari, M. (2021). Health information–seeking behavior and self-care in women with osteoporosis: a qualitative study. Archives of Osteoporosis, 16(1), 78. https://doi.org/10.1007/s11657-021-00923-8

Barcenilla-Wong, A. L., Cross, M., Fry, M., & March, L. (2020). Ambiguity hindering self-management and prevention of osteoporosis in post-menopausal women. *Archives of osteoporosis*, *15*(1), 73. https://doi.org/10.1007/s11657-020-0683-1

Barker, K. L., Toye, F., & Lowe, C. J. (2016). A qualitative systematic review of patients' experience of osteoporosis using meta-ethnography. *Archives of osteoporosis*, *11*(1), 33. https://doi.org/10.1007/s11657-016-0286-z

Beaudart, C., Silverman, S., Gold, D. T., Williams, S. A., Weiss, R., & Hiligsmann, M. (2022). A Qualitative Study to Assess US Patient Preferences between new Transdermal System and Injectable Anabolic Therapies for Osteoporosis Treatment. *Archives of osteoporosis*, *17*(1), 57. https://doi.org/10.1007/s11657-022-01075-z

Besser, S. J., Anderson, J. E., & Weinman, J. (2012). How do osteoporosis patients perceive their illness and treatment? Implications for clinical practice. *Archives of osteoporosis*, *7*, 115–124. https://doi.org/10.1007/s11657-012-0089-9

Brod, M., Rousculp, M., & Cameron, A. (2008). Understanding compliance issues for daily self-injectable treatment in ambulatory care settings. *Patient preference and adherence*, *2*, 129–136.

Dastmanesh, S., Karimi, M., Ghahremani, L., Seif, M., & Zare, E. (2023). A health communication campaign for prevention of osteoporosis in rural elderly women. *BMC women's health*, *23*(1), 124. https://doi.org/10.1186/s12905-023-02282-7

Delbari, A., Azimi, A., Najafi, M., Saatchi, M., Bidkhori, M., Mousavi, M. E., Tabatabaei, F. S., & Hooshmand, E. (2023). Prevalence, Complications, and Risk Factors of Falls and Fear of Falling Among Older Adults; Based on Ardakan Cohort Study on Aging (ACSA). *Archives of academic emergency medicine*, *12*(1), e9. https://doi.org/10.22037/aaem.v12i1.2084

Dohrn, I. M., Ståhle, A., & Roaldsen, K. S. (2016). "You Have to Keep Moving, Be Active": Perceptions and Experiences of Habitual Physical Activity in Older Women With Osteoporosis. *Physical therapy*, *96*(3), 361–370. https://doi.org/10.2522/ptj.20150131

Feldstein, A. C., Schneider, J., Smith, D. H., Vollmer, W. M., Rix, M., Glauber, H., Boardman, D. L., & Herson, M. (2008). Harnessing stakeholder perspectives to improve the care of osteoporosis after a fracture. *Osteoporosis international : a journal established as result of cooperation between the European Foundation for Osteoporosis and the National Osteoporosis Foundation of the USA*, *19*(11), 1527–1540. https://doi.org/10.1007/s00198-008-0605-3

French, M. R., Vernace-Inserra, F., & Hawker, G. A. (2008). A prospective study to identify factors affecting adherence to recommended daily calcium intake in women with low bone mineral density. *Journal of the American College of Nutrition*, *27*(1), 88–95. https://doi.org/10.1080/07315724.2008.10719679

Gül, S., & Büyükbayram, Z. (2023). "A natural consequence of aging in women?": a mixed-method exploration on osteoporosis self-efficacy in Turkey. *Archives of osteoporosis*, *18*(1), 47. https://doi.org/10.1007/s11657-023-01239-5

Hamed, K., Roaldsen, K. S., & Halvarsson, A. (2021). "Fear of falling serves as protection and signifies potential danger": a qualitative study to conceptualise the phrase "fear of falling" in women with osteoporosis. *Osteoporosis international : a journal established as result of cooperation between the European Foundation for Osteoporosis and the National Osteoporosis Foundation of the USA*, *32*(12), 2563–2570. https://doi.org/10.1007/s00198-021-06047-6

Lau, E., Papaioannou, A., Dolovich, L., Adachi, J., Sawka, A. M., Burns, S., Nair, K., & Pathak, A. (2008). Patients' adherence to osteoporosis therapy: exploring the perceptions of postmenopausal women. *Canadian family physician Medecin de famille canadien*, *54*(3), 394–402.

Lyu, F. F., Ramoo, V., Chui, P. L., & Ng, C. G. (2024). Perceptions Toward Exercise or Mindful Exercise Participation Among Patients With Primary Osteoporosis: A Qualitative Study. *Clinical nursing research*, *33*(1), 40–50. https://doi.org/10.1177/10547738231198561

Majumdar, S. R., McAlister, F. A., Johnson, J. A., Weir, D. L., Bellerose, D., Hanley, D. A., Russell, A. S., & Rowe, B. H. (2014). Critical impact of patient knowledge and bone density testing on starting osteoporosis treatment after fragility fracture: secondary analyses from two controlled trials. *Osteoporosis international : a journal established as result of cooperation between the European Foundation for Osteoporosis and the National Osteoporosis Foundation of the USA*, *25*(9), 2173–2179. https://doi.org/10.1007/s00198-014-2728-z

McArthur, C., Ziebart, C., Papaioannou, A., Cheung, A. M., Laprade, J., Lee, L., Jain, R., & Giangregorio, L. M. (2018). "We get them up, moving, and out the door. How do we get them to do what is recommended?" Using behaviour change theory to put exercise evidence into action for rehabilitation professionals. *Archives of osteoporosis*, *13*(1), 7. https://doi.org/10.1007/s11657-018-0419-7

Muhamad Jamil, N. K., Naina Mohamed, I., Mokhtar, S. A., Leong, J. F., Kamudin, N. A. F., & Muhammad, N. (2024). Barriers to osteoporosis management and adherence to Clinical Practice Guideline: a comparative study between tertiary East Coast hospitals and a Fracture Liaison Services (FLS)-accredited hospital in Malaysia. *Archives of osteoporosis*, *19*(1), 49. https://doi.org/10.1007/s11657-024-01407-1

Nilsson, C., Lindberg, B., Juuso, P., & Olsson, M. (2019). Experiences of striving to maintain daily life among women with osteoporosis. *International journal of qualitative studies on health and well-being*, *14*(1), 1647402. https://doi.org/10.1080/17482631.2019.1647402

Rezae, F., Kelly, A., Dey, S., Moles, R., & Carter, S. (2024). Healthcare professionals' perspectives and experiences of osteoporosis medication treatment: a qualitative systematic review. *Archives of osteoporosis*, *19*(1), 8. https://doi.org/10.1007/s11657-023-01359-y

Rodrigues, I. B., Armstrong, J. J., Adachi, J. D., & MacDermid, J. C. (2017). Facilitators and barriers to exercise adherence in patients with osteopenia and osteoporosis: a systematic review. *Osteoporosis international : a journal established as result of cooperation between the European Foundation for Osteoporosis and the National Osteoporosis Foundation of the USA*, *28*(3), 735–745. https://doi.org/10.1007/s00198-016-3793-2

Rothmann, M. J., Jakobsen, P. R., Jensen, C. M., Hermann, A. P., Smith, A. C., & Clemensen, J. (2018). Experiences of being diagnosed with osteoporosis: a meta-synthesis. *Archives of osteoporosis*, *13*(1), 21. https://doi.org/10.1007/s11657-018-0436-6

Saag, J. L., & Danila, M. I. (2022). Remote Management of Osteoporosis. *Current treatment options in rheumatology*, *8*(4), 143–151. https://doi.org/10.1007/s40674-022-00195-4

Salter, C., McDaid, L., Bhattacharya, D., Holland, R., Marshall, T., & Howe, A. (2014). Abandoned acid? Understanding adherence to bisphosphonate medications for the prevention of osteoporosis among older women: a qualitative longitudinal study. PloS One, 9(1), e83552. https://doi.org/10.1371/journal.pone.0083552

Schousboe J. T. (2013). Adherence with medications used to treat osteoporosis: behavioral insights. *Current osteoporosis reports*, *11*(1), 21–29. https://doi.org/10.1007/s11914-013-0133-8

Tarantino, U., Iolascon, G., Cianferotti, L., Masi, L., Marcucci, G., Giusti, F., Marini, F., Parri, S., Feola, M., Rao, C., Piccirilli, E., Zanetti, E. B., Cittadini, N., Alvaro, R., Moretti, A., Calafiore, D., Toro, G., Gimigliano, F., Resmini, G., & Brandi, M. L. (2017). Clinical guidelines for the prevention and treatment of osteoporosis: summary statements and recommendations from the Italian Society for Orthopaedics and Traumatology. Journal of Orthopaedics and Traumatology, 18(S1), 3–36. https://doi.org/10.1007/s10195-017-0474-7

Wright, N. C., Melton, M. E., Sohail, M., Herbey, I., Davies, S., Levitan, E. B., Saag, K. G., & Ivankova, N. V. (2019). Race Plays a Role in the Knowledge, Attitudes, and Beliefs of Women with Osteoporosis. *Journal of racial and ethnic health disparities*, *6*(4), 707–718. https://doi.org/10.1007/s40615-019-00569-w

Zadjali, F. A., Brooks, J., O'Neill, T. W., & Stanmore, E. (2023). Impact of postmenopausal osteoporosis on the lives of Omani women and the use of cultural and religious practises to relieve pain: A hermeneutic phenomenological study. *Health expectations : an international journal of public participation in health care and health policy*, *26*(6), 2278–2292. https://doi.org/10.1111/hex.13824

Ziebart, C., MacDermid, J., Furtado, R., Pontes, T., Szekeres, M., Suh, N., & Khan, A. (2022). An interpretive descriptive approach of patients with osteoporosis and integrating osteoporosis management advice into their lifestyle. International Journal of Qualitative Studies on Health and Well-Being, 17(1). https://doi.org/10.1080/17482631.2022.2070976

Zubick, P., & Dahlke, S. (2024). Family/caregiver influence on osteoporosis management for older people: an integrative review. *Osteoporosis international : a journal established as result of cooperation between the European Foundation for Osteoporosis and the National Osteoporosis Foundation of the USA*, *35*(7), 1153–1163. https://doi.org/10.1007/s00198-024-07081-w
