# Supplementary material for: Barriers and Facilitators to Self-Care Behaviors in People Living with Osteoporosis: A Qualitative Descriptive Study
Source: Nurs Rep. 2026 Jan 20;16(1):33. doi: 10.3390/nursrep16010033 (PMC12844863; doi:10.3390/nursrep16010033)
Supplement: Supplementary file 1 [file nursrep-16-00033-s001.zip › Supplementary S2.pdf]

Supplementary S2. Anchor samples

THEME: BARRIERS	
**Code**	**Anchor samples**
**Category: inadequate physical activity**	
Inefficient awareness	• I'm also taking vitamin D now, and I think that if I had started taking it earlier, I probably wouldn't have ended up in this situation. Back then, they didn't routinely test for vitamin D unless there was a specific reason, you know? Now I look at blood tests from 10 or 15 years ago that I still keep, and there's no mention of it at all (laughs). So, this greater attention nowadays… Well, in fact, I haven't changed my lifestyle, I've always had this one. This condition just came upon me, against my will (OP005)
Personal capability	• Well, of course, there are some things I find harder to do now, and I don't walk as much as I used to because I get tired more easily. (OP019)
Lack of exercise-related knowledge	• OK, but what about a targeted activity, like going to the gym or something like that? Right? No, no. Nothing like that. (OP007)
Low exercise self-efficacy	• I just do a bit of light exercise, like using the stairs, trying to stay active, going for walks. (OP002)<br>• OK, but what about a targeted activity, like going to the gym or something like that? Right? No, no. Nothing like that. (OP007)<br>• They also told me to do water-based exercise, but it's a bit complicated here—where would I even go for that? (OP010)<br>• I'm on my feet all day and try to move around that way, but once I get home, I'm unfortunately stuck inside. (OP011)
Lacking trust in rehabilitation workers	• …It didn't feel right to take hormones. My GP told me at the time that there was a tendency to prescribe these medications all the way to the end. So, if there's a good reason, fine; otherwise, I prefer to continue as I am. (OP006)
Lack of transportation	• To do it? (sighs) It's always about going 9 kilometers out. (OP010)
Uncertainty	• I'm still deciding whether to take it or not… I sensed a certain carelessness around it, but this is a drug that can cause problems for people with heart conditions, so I'm trying to understand a bit more before making a decision. We'll see. (OP001)
Fear of falling	• I'm afraid something might happen, like fractures… I'm scared of the consequences when doing other things. (OP002)<br>• You know, I try to be careful, because with osteoporosis you have some pain and swelling, and you need to be cautious—especially about falling. For me, it's worsened a bit in the hip. (OP004)<br>• I've only done very, very gentle activities, because I'm aware that I could fall or something else could happen. For example, when I get up suddenly from a chair, I feel stiffness before I can start walking again. I feel I've lost that elasticity, that fluidity of movement. And I feel it strongly—it puts me on alert, you know, because I fear the risk of falling or tripping. (OP017)
**Category: ineffective self-efficacy**	
Knowledge gaps	• I sensed a lot of carelessness behind it, but this is a drug that can cause problems for people with heart conditions, so I'm trying to look into it a bit more to understand better. We'll see. (OP001)

	• So I don't know if it's due to the osteoporosis or, as I said before, it might just be regular pain…Sometimes you think it could be arthritis, or maybe not. But it could also be a consequence of osteoporosis, so I'm not sure if it's that or something else, you know? (OP002)<br>• What I absolutely can't give up is milk. They told me not to overdo it, but if I don't have my cup of coffee with milk in the morning, I just don't feel right. (OP003)<br>• I'm also taking vitamin D now, and I think that if I had taken it earlier, I probably wouldn't be in this situation. Back then, they didn't check vitamin D levels unless it was specifically requested, right? But now I see that… (OP005)<br>• Who suggested the behaviors to help keep your osteoporosis stable? No one. (OP006)<br>• No, no one suggested it to me. (OP013)<br>• Not really. I don't know much about it... I'm not someone who goes online to look up how to treat these things. (OP015)<br>• The doctor told me, "Well, since they gave you too much calcium—more than you needed—your bones became too hard. Blood didn't circulate properly anymore, and that caused that lesion in the center." (OP018)<br>• Honestly, I don't know anything. I just know I need to be careful not to fall. What else can it do to me? I don't know. (OP019)<br>• I don't think cholecalciferol (vitamin D) causes any issues, even though I haven't read the leaflet, so I'm not particularly informed. (OP020)
Disease self-perception	• No, I don't really feel anything. I don't feel the osteoporosis, but I know it's there from the charts and lab values. It's not something that affects me physically... Honestly, I don't even know where it's coming from anymore—maybe it's just in my head. (OP001)<br>• So I don't know if it's due to the osteoporosis or, as I said before, it might just be regular pain… Sometimes you think it could be arthritis, or maybe not. But it could also be a consequence of osteoporosis, so I'm not sure if it's that or something else, you know? (OP002)<br>• I don't know if this curling in on myself is due to osteoporosis or something else. Objectively, I don't know if it's a matter of arthritis or osteoporosis. (OP003)<br>• The negative part is having to go through all these treatments, especially when you know you need to go for injections with the orthopedist. (OP004)<br>• They told me that it's not actually something serious at the moment, so I don't really perceive it as a disease. (OP005)<br>• I monitor it when I get a DEXA scan. Let's say… No, I haven't had any problems. How can I monitor it if it changes? These are things that progress so slowly that we don't even notice. (OP006)<br>• I go walking while on the phone so I don't think about anything (laughs), and that way I don't feel anything. (OP007)<br>• I don't know how to assess it. To me, this treatment with denosumab feels more like an experiment, to be honest. (OP013)<br>• You don't feel anything, you know? No pain from anywhere—I don't feel pain. But does osteoporosis even cause pain? (OP015)<br>• Since I don't really like going to the doctor, I'm not the kind of person who's very diligent about check-ups… So at first, it was a bit like I didn't really recognize myself in all this. (OP017)

	• No, I wouldn’t say there’s any real risk… other than feeling tired in the legs, nothing else. I don’t notice anything. (OP018)<br>• Do you have any sensations that make you feel like something might not be going well with your osteoporosis management? Um, I’d say no. (OP020)
Disease social-perception	• I find it a harsh, not very reassuring way… because I think it affects one’s appearance, the physical aspect…But there's this feeling of insecurity… of instability, of unease. It really affects the overall experience, you know, all the problems that each of us might have… (OP005)<br>• It could be a kind of disability—not a real one, but the beginning of a future disability. It could be, depending on how well it’s treated. (OP010)<br>• Free from the pain that was always lurking, ready to say: “No, you can’t do that. No, you can’t. You’re unwell. You need to send a doctor’s note.” For me, it was something… (OP016)<br>• So at first, it felt like I didn’t quite recognize myself in this condition… and so mainly it was a moment of realizing that I might be someone starting to have problems related to… well, to aging. There was a phase of acceptance. (OP017)
Aging	• The cartilage in my knees has worn down. That’s logical, because I’m also at an age where that’s entirely possible. (OP012)<br>• Of course, at seventy years old, I can’t exactly take up a sport. (OP013)<br>• So at first, it felt like I didn’t quite recognize myself in this condition… and so mainly it was a moment of realizing that I might be someone starting to have problems related to… well, to aging. There was a phase of acceptance. (OP017)<br>• Gradually, over the years, there’s been more and more tiredness in my legs. (OP018)<br>• The degeneration is faster now, because, well… we’re getting old (laughs), if we aren’t already. (OP020)
Not exercising regularly	• I should do exercise and move more—I know I should—but I don’t... I’m lazy, inconsistent, and generally unreliable in everything, including taking care of my body. (OP001)<br>• I just do a bit of light exercise, like using the stairs, trying to stay active, going for walks. (OP002)<br>• Yes, I walk, but they told me not to overdo it. (OP004)<br>• Nothing really—I do the grocery shopping, stay at home, go around a bit, that kind of stuff. I don’t do anything (laughs). (OP007)<br>• I work, I go to work and that’s it. Then I take care of everything else I have to do. (OP011)<br>• To tell you the truth! (laughs loudly) Every 15 days we’re there, unless there are other appointments—so yes, plenty of activity! Anyway, I used to do exercise, honestly: I used to go to the gym, do workouts, but now I’ve stopped everything… I should do some exercise. (OP012)<br>• During the pandemic, I actually stopped working, so that was also a problem for me… a moment to reassess some lifestyle habits which before included more movement, going out, doing more physical activity. (OP017)

Inadequate consumption of milk and dairy products	• I should watch my diet… None. I’ve had no appetite since birth—I eat very little, and poorly. (OP001)<br>• What kind of diet are you following for your osteoporosis? None. (OP002)<br>• What I absolutely can’t give up is milk. They told me not to overdo it, but if I don’t have my cup of coffee with milk in the morning, I just don’t feel right (laughs). (OP003)<br>• Calcium, yes, but lately—for more than a year now—milk has started bothering me, so I’ve stopped having cappuccino for breakfast because it upsets me. (OP004)<br>• I’m supposed to avoid cheese because of my cholangitis, but for osteoporosis I should be eating it. So instead of drinking milk in the morning, I have soy milk, and at lunch maybe a nice piece of Parmigiano Reggiano. That’s how I try to balance things. (OP008)<br>• No, I eat normally, as I always have. (OP013)<br>• Not for osteoporosis, no—even though it probably conflicts with my cholesterol diet, since my cholesterol is a bit high. (OP015)<br>• Are you following a specific diet for osteoporosis? No. (OP018)<br>• No, absolutely not—I eat whatever’s around (laughs)… not great. (OP019)<br>• No, the Mediterranean diet. (OP020)
Inadequate exposure to sunlight	• I try to get more sun, but I can’t stay in the sun too much because of my pacemaker. (OP004)<br>• I sit in the sun when I get the chance, but I don’t go to the beach very often. (OP006)
Being a caregiver For others	• My husband isn’t well either (sighs). (OP012)
Lack of support people/caregivers	• I don’t have anyone supporting me in that sense. (OP005)<br>• It ruined me in the sense that I couldn’t say anything to anyone anymore. So I stopped taking everything, because I really couldn’t talk. (OP010)<br>• I still had to take care of my husband, because everything happened during COVID. He had a stroke, so I was more focused on him than on myself… (OP011)<br>• My husband isn’t well either (sighs)... I need to have someone who can help me. (OP012)<br>• I shouldn’t go too far, and it’s not always easy to call a friend and say, “Oh God, I’m not feeling well,” because I’ve never really leaned on others… you know? (OP014)
Inadequate unhealthy diet	• None. I’ve had no appetite since birth—I eat very little and poorly… I eat very little, at odd hours, only when I get a stomachache, because I don’t feel hunger. (OP001)
Inefficient awareness	• My primary care doctor keeps telling me, and so does my trusted pharmacist (laughs), so it’s not that I don’t have the tools—I just don’t use them. (OP001)<br>• What I absolutely can’t give up is milk. They told me not to overdo it, but if I don’t have my cup of coffee with milk in the morning, I just don’t feel right. (OP003)

	• So, there should be a more targeted campaign focused on this disease. (OP004)<br>• Oh God, I don't remember. Maybe they did tell me something, but… I didn't pay attention. (OP007)<br>• Otherwise, I wouldn't have even known. So next time I go to the dentist, I'll say, "Look, I'm undergoing treatment," and explain everything. Now that it's closed… before, I didn't know. (OP012)<br>• You don't feel anything, you know? No pain from anywhere—I don't feel pain. But does osteoporosis even cause pain? (OP015)<br>• So at first, it felt like I didn't quite recognize myself in this condition… and so mainly it was a moment of realizing that I might be someone starting to have problems related to… well, to aging. There was a phase of acceptance. (OP017)<br>• No, I don't do anything specific. I'm active in the morning and evening: I have grandkids, I take them to school, pick them up… I drive, but I also move around a lot at home—I'm never still. (OP018)<br>• But I don't… I don't ask myself questions like, "Oh God, should I…?" I just try to be careful—when walking, when doing things—because they told me from the DEXA scan that I'm at risk of fractures, so I'm cautious. (OP019)
Self-neglect	• Because of a general lack of care—not just related to osteoporosis… I don't take care of myself… not smoking (the patient lights a cigarette) … It gave me an excuse not to run, not to walk, not to exercise. It became a justification. (OP001)<br>• I haven't changed my lifestyle. (OP006)<br>• Are there any other things you're doing for your osteoporosis? No. (OP007)<br>• I got tired of it; so I stopped, and then I didn't… I didn't do the DEXA scan as regularly as I should have. (OP015)
Personal capability	• I can't take care of myself… I've had problems with my leg, I had a thrombosis, and after walking for a while, I feel strong fatigue in my left leg. So I put more strain on the right one, and then the right one gets tired too. (OP001)<br>• Like, yes, swimming is good too, but I shouldn't stay too long in the water… because I can't do too much—then I get tired, both in terms of my heart and my hip. If I do too many exercises, I get tired. (OP004)<br>• Lately, I find it quite exhausting (laughs). So, it's a bit limiting, all things considered, right? It lingers... (OP005)
**Category: difficulties in osteoporosis management**	
Side effects	• Because I drink a lot, I urinate a lot, but sometimes I'm out and about and don't have access to a bathroom. (OP001)<br>• The only thing is the calcium—I take it in soluble sachets mixed with water, and it was bothering me. (OP004)<br>• Fosamax has to be taken on an empty stomach, staying upright because it can cause problems if you have gastritis. So I adapt—I adjust. (OP006)<br>• This stuff, this thing here… It's just that I have a bit of a throat issue, so when I take the pill, I start going "UHM" (throat noises), you know what I mean? But I already know—it's a problem I have—but I don't feel those shooting pains, this pain thing, you understand? (OP007)<br>• After a while, there was that injection you have to take daily. I started feeling cramps, mainly in my legs, and so they stopped it for me. (OP008)

	• I react with side effects (sighs). I'm sensitive—too sensitive to certain things. So instead of benefiting (sighs), it might help with one thing but cause five or six other side effects elsewhere. (OP010)<br>• I've also taken other medications for osteoporosis, and they bothered me. Now, since I've been getting this last… this injection every six months… and as I've already told you, I also take cholecalciferol regularly… things are a bit better, though now I've read that… sometimes my teeth bother me. (OP012)<br>• If the pain is unbearable, the only thing that helps is a corticosteroid injection—but even there, we have to be careful, because apparently corticosteroids worsen osteoporosis. (OP013)<br>• The results weren't noticeable, because the medications you take might help one thing but worsen others, like the liver—or they change your metabolism a bit. Some of those changes might be worse than osteoporosis itself. So at that point, I took it slow. (OP014)<br>• Also because I had stomach issues—at one point, reactive gastritis and so on. (OP016)<br>• So one thing I've noticed is a bit of stomach pain. (OP017)
Bad Relationship with healthcare providers	• I sensed a lot of carelessness behind it, but this is a drug that can cause problems for people with heart conditions, so I'm trying to look into it a bit more to understand better. We'll see. (OP001)<br>• Well, we try—if necessary, the doctor… I don't know, maybe he'll run some tests. I always follow my primary care doctor's advice first. He might say: "I don't know, let's do some tests, repeat the vitamin D, we'll see." Or he might say: "Go see the orthopedist." (OP004)<br>• After a couple of years, they basically kicked me out because I said (laughs) that I wasn't following what they told me, and they needed to make room for someone more willing to stick to their program. So I left. (OP006)<br>• No one… They didn't... I went there to be examined and they just showed me this chart and said, "Go get treated so you can recover." (OP007)<br>• It's not like there's a doctor here you can just call and they come. We used to pay someone before... so they would come. Now, maybe they're available—or you can only call at a specific time they set. It's all complicated… that's why. (OP010)<br>• Yes, yes, I changed doctors because the first one—she didn't satisfy me at all. (OP011)<br>• But when someone goes to a visit, at least a little… I'm not saying you should stay in there for half an hour, God forbid—but at least a *minimum* (pronounced slowly)—a minimum amount of information should be given. Like: "Be careful with this, be careful with that" (spoken with dialect). "Avoid doing this." Nothing at all. Just "How are you?" "Still the same." I have to tell them everything: "I have this, I have that." And they barely respond. So what's the point? (OP012)<br>• At the hospital where I went, Tor Vergata, I always saw students. I *never* (emphasizes the word) had the chance to speak with the actual head of the trial. Every time, it was different students. (OP013)<br>• If it were considered a disease to be prevented like other conditions, that wouldn't be bad—I'd feel more supported. Instead, I'm guided by my own instincts and personality, not by… not by the healthcare system. (OP015)<br>• Since I don't… I don't like going to the doctor much, I'm not the kind of person who keeps up with check-ups… so at first, it felt a bit like I didn't recognize myself in this. (OP017)

	• My GP never tells me anything about this osteoporosis because it's not her field; I'm under follow-up there, I just bring the medication list, and she writes the prescriptions for me… (unclear speech) I don't even see the doctor. (OP018)<br>• I think it was the head of the department who told me that if I wasn't planning to follow their… their instructions, there was no point in coming—because I had to make space for someone who was more interested than I was. And I said, "Well, whatever, who cares." (OP020)
Conflicting advice	• No, no, because, as I already told you, I suffer from many conditions, so when I feel something's wrong, I don't know which illness to blame. When I talk to the osteoporosis specialist, they say "It's not from that." When I talk to the liver specialist, they say "No, it's not that either." When I (laughs) talk to the pulmonologist, they say "Not from that"… So do I have yet another illness? (laughs) Let's just forget it—I'm fed up. (OP012)<br>• Because I had an appointment at my local health unit and found someone who probably didn't even know how this medication works. Many don't know about it and advise against using it. (OP013)<br>• And not having a point of reference—because for us patients, if you'll allow me the term—having one clear, consistent reference point is extremely important. (OP016)<br>• If they saw that my calcium levels were fine, they should have reduced it… that kind of monitoring should have been maintained. (sighs) I followed what they told me—I took a lot of calcium when they said to. They should have either increased or reduced it based on the test results. (sighs) I just hope they did. (OP018)<br>• And my doctor said, "Look, knowing you and your lifestyle, I advise against it because it might just be a case of overprescribing medication." (OP020)
Disease-related symptoms	• I'm someone who moves in sudden bursts—I'm always quite restless, and this hasn't improved my way of being. I still move in a jerky way. (OP001)<br>• Lately, I find it a bit tiring (laughs). So it's a bit limiting, all in all, right? It lingers… Yeah. (OP005)<br>• Like here, in the hip… mainly here—this is what hurts when I walk. (OP010)<br>• I crack all over—I literally feel my bones going "crack crack." It's annoying, unpleasant. It doesn't hurt, but it bothers me. (OP011)<br>• I said I've been having more frequent cramps… I already had them, but only rarely. Now they're more frequent, so all I can do is report it. (OP012)<br>• If the pain is unbearable, the only thing that helps is a corticosteroid injection—but even there, we need to be careful, because corticosteroids are said to worsen osteoporosis. (OP013)<br>• Yes, yes—I felt a heaviness in my head. It felt like a big wad of cotton around it. I know myself, and that's important. (OP014)<br>• The limitation—functional limitation—that pain causes leads me to having to take medication, of course. (OP016)<br>• Definitely a bit of stiffness… during the change of seasons, or when the weather is particularly damp, I feel a kind of stiffness. A lack of fluidity—that's the sensation. (OP017)

	• Fatigue—because at some point, hour after hour… doing this, doing that, never stopping (unclear speech), one thing after another for five or six hours straight… It’s normal to feel a bit tired. (OP018)<br>• No, no—you know when it hits me? When I’m lying in bed, resting, or sitting in a chair. When I get up, I have trouble getting moving again—I feel more pain. But as I start moving again, the pain eases. Well, of course, some things are harder to do now, and I don’t walk as much as I used to because I get tired more quickly. (OP019)
Difficulty accessing care	• This year, trying to get an appointment for the DEXA scan and the consultation was a nightmare—an absolute nightmare. I booked it in December and got in by March because I just couldn’t get an appointment. (OP001)<br>• I think that, at least in my case, it would be helpful—especially since I have other health issues. (OP002)<br>• I booked the ultrasound, but I also want to do one privately, because I’d rather get it done sooner than wait until July or November. So that’s what I’m doing. If follow-ups were closer together, like I said that day, I’d be happier. But since they decide things that way, I manage it on my own with a private orthopedist. (OP004)<br>• I'm also taking vitamin D now, and I think that if I had taken it earlier, I probably wouldn't be in this situation. Back then, they didn’t check vitamin D levels unless it was specifically requested, right? But now I see that… (OP005)<br>• No one… They didn’t... I went there to be examined and they just showed me this chart and said, “Go get treated so you can recover.” That’s it. (OP007)<br>• It’s not like there’s a doctor here you can just call and they come. We used to pay someone before so they would come. Now maybe they’re available—or you can only call during a specific hour they’ve set. Everything is complicated… that’s why. (OP010)<br>• When I went to Tor Vergata, I told them the first time—I mentioned my knees, I mentioned the cramps. They told me I needed to go to the… to the… what’s it called? (sighs) Basically, they made it clear that it had nothing to do with osteoporosis. So what should I do? They said I had to see the orthopedist, that I had to get X-rays, because apparently there's… something else. (OP012)<br>• The downside is that I had to get a removable prosthesis instead—I couldn’t get an implant. (OP013)<br>• In the end, I called, but the earliest appointment was “when pigs fly” (dialect expression), so I paid for it privately. Basically, there’s no priority lane—even if you’re a certified osteoporosis patient, there’s no advantage, no fast track for DEXA scans or anything else. (OP015)<br>• The lack of care and attention could make this condition more disabling. Not having a point of reference would definitely be a barrier. (OP016)<br>• Diagnosis times, definitely—diagnostic tests should be more accessible. (OP017)<br>• I think it was the head of the department who told me that if I wasn’t planning to follow their instructions, there was no point in coming—because I had to make space for someone more interested than me. And I said, “Well, whatever, who cares.” (OP020)
Non-adherence to therapy	• I should be more careful about taking my medications. (OP001)<br>• No, no—I stopped taking it. I stopped because I noticed that it was what was making me feel heavy. (OP004)<br>• They prescribed me hormones, but after talking to my GP, I refused to take them. (OP006)

	• I try not to forget it, because at the beginning it wasn't easy—even though it's an effervescent tablet, remembering to take it was a bit traumatic. (OP011)<br>• Honestly, I try to be a bit careful, even if I sometimes forget. (OP012)<br>• Then I stopped the treatment because, you know, living with this fear that the treatment itself might make things worse… (OP014)<br>• I stopped treating myself. He told me, "Do it for one more year," and it was during the pandemic—but after 3 or 4 months, I got fed up and quit. (OP015)<br>• So, the doctors there wanted to give me hormones, but I was pretty, let's say… skeptical. (OP020)
Gaps in prevention	• Yes, I walk, but they told me not to overdo it. (OP004)<br>• OK, but what about a targeted activity, like going to the gym or something like that? Right? No, no. Nothing like that. (OP007)<br>• When it comes to taking medicine, I'm a bit opposed. (OP008)<br>• None, none at all—because I haven't changed anything. (OP009)<br>• But the side effects were what they were, and I wanted to stop, but these are the medications available now. (OP010)<br>• I don't know how to assess it. To me, this treatment with denosumab feels more like an experiment, to be honest. (OP013)<br>• You tend to undergo… more check-ups, let's say. (OP015)<br>• No, because if you feel fine, then… (sighs) what for? What could they even give me? For me it's always the same. It's not like I say, "Oh God, I've got this, and if I take that, I'll feel better or worse"—no, for me it's all the same. I don't feel any symptoms. (OP018)<br>• So, the doctors there wanted to give me hormones, but I was pretty, let's say… skeptical. (OP020)
Uncertainty	• So for me it's hard to tell—I've always had intestinal issues, and my stomach… I've had this constant feeling of nausea for years now, so it's not easy for me to understand. (OP001)<br>• I don't know if there's another way, but I read that this is the only option—other things are better avoided. I knew about this new treatment, but I know it's not really an option. (OP002)<br>• I don't know if this curling in on myself is due to osteoporosis or something else. Objectively, I don't know if it's a matter of arthritis or osteoporosis. (OP003)<br>• I don't know if I'm managing the disease well, but I try to go with what I feel—based on intuition, you know? (OP005)<br>• Nothing—what can I do? I don't know if there's some remedy or something you can do… I really don't know, you know? (OP007)<br>• There could be contributing factors that… you know… (OP010)<br>• What do you know about osteoporosis and its possible complications? Well, almost nothing. (OP011)<br>• Whether they're related to one thing or not… I don't know. (OP012)<br>• I don't know how to assess it. To me, this treatment with denosumab feels more like an experiment, to be honest. (OP013)<br>• You don't feel anything, you know? No pain from anywhere—I don't feel pain. But does osteoporosis even cause pain? (OP015)

	• And not having a point of reference—because for us patients, if you'll allow me the term—having one clear, consistent reference point is extremely important. (OP016)<br>• This whole situation gives me a sense of insecurity and instability. (OP017)<br>• But I always put a lot of trust in them, and I ended up needing surgery… I fell down… So now there's this big question mark. (OP018)
Impact in daily routine	• Not being able to have breakfast right after waking up—having an empty stomach—really bothers me. (OP001)<br>• There are definitely some limitations—not so much health-related, but because, well, you have to follow the treatment, take the medication, and be careful. (OP004)<br>• Lately, I find it a bit tiring (laughs). So it's a bit limiting, all in all, right? It lingers… Yeah. (OP005)<br>• Well, the hassle (laughs) of having to wake up early once a week and stay upright—because I'm quite lazy. (OP006)<br>• I basically had to do a daily injection, and that was a bit more of a struggle. Having to take the medication with you everywhere—especially on vacation—with the cold pack and all, that was a bit annoying. (OP008)<br>• Eh, I don't know—I try to do what they tell me. (OP009)<br>• The difficulty of… (sighs) being limited. I used to ride my bike a lot, you know? Now I can't anymore. (OP010)<br>• I try not to forget it, because at the beginning it wasn't easy—even though it's an effervescent tablet, remembering to take it was a bit traumatic. (OP011)<br>• I need to have someone who can help me. (OP012)<br>• The downside is that I had to get a removable prosthesis instead—I couldn't get an implant. (OP013)<br>• Feeling that chronic pain causes discomfort—you can't manage your social or work life anymore. (OP016)<br>• I've only done very, very, very gentle activities, because I'm aware that I could fall or that something else might happen to me. (OP017)<br>• Well, of course, some things are harder to do now, and I don't walk as much as I used to because I get tired more quickly. (OP019)
Comorbidities	• So for me it's hard to tell—I've always had intestinal issues, and my stomach… I've had this constant feeling of nausea for years now, so it's not easy for me to understand. (OP001)<br>• I can't do that because I have a pacemaker, so I can only do local treatments… like, I mean, I can't do ultrasound therapy. I can only get massages, and I can do mud therapy, but the mud can't be too hot either. (OP004)<br>• I'm supposed to avoid cheese because of my cholangitis, but for osteoporosis I should be eating it. So instead of drinking milk in the morning, I have soy milk, and at lunch maybe a nice piece of Parmigiano Reggiano. That's how I try to balance things. (OP008)<br>• What I have is mostly rheumatic problems—they're what cause me pain. For example, my neck gets inflamed, my back hurts—those kinds of things. So I do regular checkups for osteoporosis, but my main issue is probably more rheumatologic. (OP009)<br>• I have to be careful because I also suffer from primary biliary cirrhosis, so I need to watch out for many things. (OP012)<br>• Not for osteoporosis, no—even though it probably conflicts with my cholesterol diet, since my cholesterol is a bit high. (OP015)

Osteoporosis being overlooked by other specialists	• The cardiologist told me that if they got too large, yes, it could damage not only the heart but also the circulation. And since I'm not young anymore, the weight could also become a burden. (OP003)<br>• So, there should be a more targeted campaign focused on this disease. (OP004)<br>• I'm also taking vitamin D now, and I think that if I had taken it earlier, I probably wouldn't be in this situation. Back then, they didn't check vitamin D levels unless it was specifically requested, right? But now I see that… (OP005)<br>• Who suggested the behaviors to help keep your osteoporosis stable? No one. (OP006)<br>• But they didn't notice anything. (OP018)<br>• Well, no… As far as diet goes, I've gone to the dietitian several times because I'm slightly overweight. But I try to eat well—I try to avoid sweets, even though I have a sweet tooth… (OP020)
Disagreement with osteoporosis treatment	• The negative part is having to go through all these treatments—especially when you know you need to get injections from the orthopedist. (OP004)<br>• In my opinion, the treatments are just palliative. They don't really address the root of the problem—they're not curative. (OP013)<br>• So, the doctors there wanted to give me hormones, but I was pretty, let's say… skeptical. (OP020)
Financial constraints	• But there are people who don't even have the means to buy medicine. For example, I've also taken supplements—and they're all out of pocket! (OP004)<br>• If you can't afford it… (sighs) Right now, I'm going through a rough patch, so I didn't go to the doctor—I just wrote to him, and he kindly replied. (OP015)
Cost of illness	• Well, the hassle (laughs) of having to wake up early once a week and stay upright—because I'm quite lazy. (OP006)<br>• I basically had to do a daily injection, and that was a bit more of a struggle. Having to take the medication with you everywhere—especially on vacation—with the cold pack and all, that was a bit annoying. (OP008)<br>• Well, I was a bit traumatized, honestly—I never expected something like that. (OP011)<br>• The downside is that I had to get a removable prosthesis instead—I couldn't get an implant. (OP013)<br>• If you can't afford it… (sighs) Right now, I'm going through a rough patch, so I didn't go to the doctor—I just wrote to him, and he kindly replied. (OP015)<br>• And feeling that pain becoming chronic—it creates a deep discomfort. You're no longer able to manage your social life or your work life. (OP016)<br>• It affected me negatively—both physically and psychologically. (OP017)<br>• Well, of course, some things are harder to do now, and I don't walk as much as I used to because I get tired more quickly. (OP019)
Lack of disease prevention services	• I'm also taking vitamin D now, and I think that if I had taken it earlier, I probably wouldn't be in this situation. Back then, they didn't check vitamin D levels unless it was specifically requested, right? But now I see that… (OP005)<br>• But if I had known earlier—"look, you need to be more active, do more things"—it all felt a bit uncertain. (OP010)

	• They told me, "Look, there's no need to do anything anymore, just take some vitamin D, that's enough." But then, 2 or 3 months ago, I took it to my doctor and he said: "Wow, this is really bad. At least did the specialist prescribe you medication?" And I said, "Actually, no." (OP011)<br>• For this kind of condition, there's nothing planned—no preventive care—especially for us women, who suffer more from it. It's still not seen as a disease that needs to be prevented. In my opinion, if you intervene early—I mean, (sighs) like in my case, I've improved, but it was all on my own. (OP015)<br>• We absolutely need to open ourselves to the path of prevention, prevention, prevention, prevention. We have to adopt a mindset and a system of education—education is fundamental to prevention. We need to catch it before the symptoms appear, when possible. We must get informed. General practitioners must take care to inform women of my age—and even younger. (OP016)<br>• It's definitely also a prevention issue. (OP017)
**Category: ineffective coping strategies**	
Lack of Coping skills	• I'm lazy, inconsistent, and unreliable in general—not just when it comes to taking care of my body. (OP001)<br>• Well, the hassle (laughs) of having to wake up early once a week and stay upright—because I'm quite lazy. (OP006)<br>• That's mainly my problem—and then this creates anxiety. I try to do it, but I'm anxious, so I don't know… Now, for example, I have to give myself these injections, and I think, "What if I do it wrong? What if I hurt myself?" (sighs) (OP010)<br>• "I leave this room and go to the other one where the doctors are, and they tell me what to take—and I take it." I don't really know how the medications were managed… it's like that. (OP018)<br>• But I don't… I don't ask myself questions like "Oh God, should I…?" I just try to be careful—when walking, when doing things—because the DEXA scan says I'm at risk of fractures, so I'm cautious. (OP019)<br>• And I said, "Well, whatever, who cares." (OP020)
Psychological Distress	• I also get anxious… there it is (sighs). This whole situation creates anxiety, so you're never really at ease. (OP010)<br>• I honestly didn't feel good about it (laughs), because I'm not that old—I mean, I'm older, but not so old that I should already need a prosthesis. (OP013)<br>• The one in the spine caused me a lot of anguish (sighs)… I rushed to get treated because the idea of a spinal fracture was unacceptable to me. I would have taken it very badly. I mean, I don't know exactly what the consequences are, but I can imagine. It has happened in my family too, sort of… (OP015)<br>• Work-related stress—stress in general. (OP016)<br>• From a physical limitation perspective and also, let's say, psychologically—because it coincided with a time when I had to rethink certain things in my personal life… it was very negative. (OP017)

Fear of falling	• I'm afraid that with one of my sudden or exaggerated movements, I might break something. (OP001)<br>• But yes, there's the fear of walking. (OP008)<br>• I'm always afraid, you know? Since I have a femur at risk of fracture, I'm afraid of falling… afraid of falling and hurting myself. (OP011)<br>• Now? Now I'm terrified. So when I walk down the street, I'm very careful—because I'm terrified now. (OP012)<br>• The problem is if you fall. That's the real risk. If I were to fall… (sighs) yes, I won't lie—I'm scared. So to avoid that, I try to be cautious. (OP014)<br>• This situation gives me a sense of insecurity and instability. (OP017)<br>• I must not fall. I have to be careful, because otherwise I might be at risk of fractures… but I don't ask myself questions like "Oh God, should I…?" I just try to be careful—when walking, when doing things—because the DEXA scan says I'm at risk of fractures, so I'm cautious. (OP019)
Fear of fractures	• Spontaneous fractures—that scares me… I'm afraid that with one of my sudden or exaggerated movements, I might break something. (OP001)
**THEME: FACILITATORS**	
**Code**	**Anchor samples**
**Category: Osteoporosis management after a fracture**	
Testing after a fracture	• The DEXA scan showed there was a decrease, so they sent me to the osteoporosis center. (OP013)<br>• I repeated the DEXA scan and saw that my values had dropped. (OP014)<br>• I'm fairly disciplined, so in the end, I still go through with these checkups. (OP015)<br>• Anyway, I do screenings. I mean, I get a DEXA scan every six months. (OP016)<br>• I did it after about a year, and already in the first DEXA scan there were signs. (OP017)<br>• Women, as soon as they enter menopause, definitely need to get checked… so the DEXA scan, bone densitometry. (OP017)<br>• "They told me: 'You have significant osteoporosis. Do you know how bad it is? Are you under anyone's care?'" And I said, "I leave this room and go to the other one where the doctors are, and they tell me what to take—and I take it." (OP018)<br>• Of course, you have to get regular checkups and take the medication they prescribe—because vitamin D helps, and that little pill—I don't know if it's calcium or what—helps too. And then you have to be careful not to do anything… dangerous. (OP019)<br>• So I do take care of myself… not just for osteoporosis—my goal is more general, not specific to osteoporosis. (OP020)
Informing after a fracture	• So it's been about ten years that I've been receiving treatment… but from what I've read, these are the things they prescribe. (OP013)<br>• I read up on a lot of things, so I'm quite proactive. (OP014)<br>• I've informed myself a lot—also because I enjoy it… Primary care doctors must make sure to inform women. (OP016)
Tailored education after a fracture	• Many don't know about it and advise against doing it. But I know how to evaluate where the advice is coming from. (OP013)
Support after a fracture	• Knowing that we're not alone. (OP016)

Orthopedic advice after a fracture	• Femoral and lumbar bone densitometry, and then annual blood tests, just like the doctors tell me to do. (OP008)<br>• Of course I do the treatments. When I fell, they put a cast on me, then I wore a brace, and then I did therapy. Of course, of course. (OP012)<br>• Sure, who am I supposed to turn to? It's not like I'm going to see a guru or a priest. Always the doctor (laughs), obviously. Maybe I could use a different approach, talk to someone else… But even among orthopedists, not everyone agrees on this type of treatment! (OP013)<br>• …As soon as I saw the DEXA scan, I immediately wrote to the doctor and said, "Unfortunately, I'm slipping backward a bit," and he tried to reassure me. (OP015)<br>• So, the DEXA scan I had in April showed a T-score result of more than -2.5… And, well, that's an objective value. (OP017)<br>• "They told me: 'You have significant osteoporosis. Do you know how bad it is? Are you under anyone's care?'" And I said, "I leave this room and go to the other one where the doctors are, and they tell me what to take—and I take it." (OP018)
**Category: Osteoporosis control**	
Continuity	• Once a year I get a DEXA scan and a check-up at the orthopedics department at Tor Vergata. That's the only attention I really give to this osteoporosis. (OP001)<br>• Appointments, check-ups—those things I have to do annually, monthly, or every six months—I follow my doctor's instructions. (OP002)<br>• Once a year I go to the orthopedist, once a year to the gynecologist, once a year to the cardiologist, and once a year to the pulmonologist. So I do these check-ups and follow up based on the results. (OP003)<br>• To my GP? Well, when I notice some issues… Other times I call him, and sometimes he says: "I don't know, get these tests done and bring them to me." So for example, this test—the orthopedist at PTV told me, "It's a bit off, talk to your doctor and get further tests." They told me to get a thyroid ultrasound and more bloodwork, and my doctor said, "Yes, let's do more testing." So when I have doubts, I talk to him—he's very available. If needed, I call and we make an appointment. He says, "Now everything's by appointment" (unclear). I go in person, bring him the results, and he tells me, "Do this, do that." (OP004)<br>• I've entered the osteoporosis care circuit, so that allows me to monitor things and stay up to date. (OP005)<br>• The hospital has its own scheduling and they call me when it's time. (OP010)<br>• Anyway, my main point of reference is always my doctor. (OP013)<br>• I get checked every 18 months—so more or less every year and a half. I sent the DEXA scan in, and at first they said, "When it looks like this, don't worry." (OP015)<br>• And in that regard, we were very happy. Because what happens is that yes, the professional matters—but the communication and participation of these amazing people helped us a lot. They really put us on the right path. They're communicative and truly exceptional professionals. Honestly, it's really helpful to have these kinds of professionals available to us. (OP016)<br>• I wanted to make a comment about the osteoporosis clinic at Tor Vergata, because I think that these are chronic conditions, and it's important that healthcare services take responsibility and are aware of that. (OP017)<br>• I've always done DEXA scans and kept up with check-ups since I turned 50… I've been going for a long time. (OP018)<br>• I check in once a month… I've got it all organized (laughs). For osteoporosis, I go only to the osteoporosis specialist. (OP019)<br>• I used to show up every year for the DEXA scan and a check-up… Now I do it once a year—the scan and the consultation. (OP020)

Mutual help	• I say, "Do this, do that." We talk—she gives me advice; I give her advice. (OP004)<br>• Maybe this attention also comes from seeing my mother, because this disease was disabling for her. (OP005)<br>• By listening to my friends. (OP011)<br>• And I've always had many people around me—associations, professionals, friends… (OP014)<br>• The young people I mentor in external projects need someone who's always present, and so I love taking care of myself in that sense…My sister and I do these things together, because we both have the same condition. In a way… it's something very familiar. That's an example—we both… (OP016)<br>• My husband comes with me wherever I go, at any time, so I don't have any issues related to osteoporosis—I don't ask anyone else for help, let's say. (OP019)
Peer support	• Well, since another friend of mine does it, that's good enough for me. (OP011)<br>• It happened to me… with a couple of friends, I told them that I recommended—really promoted—that there are treatment plans at the hospital, there are doctors, orthopedists or gynecologists who can prescribe a medication. (OP014)<br>• I started getting DEXA scans only because I knew that all the women in my family were affected—so… (OP015)<br>• And my sister, yes, unfortunately… (sighs) she's also affected by this condition. So I kind of felt obliged to go get some tests done. (OP016)<br>• I can tell them what happened to me—like I did where I go for physical therapy. I said, "This is how things were, and this is how they are now." (OP018)<br>• My husband comes with me wherever I go, anytime—and I don't have any issues related to osteoporosis. I don't ask anyone for help, really. (OP019)
Good Relationship with healthcare providers	• Would you ask the doctor to change the medication? Yes, probably yes—but… (OP001)<br>• I would immediately consult my family doctor—he would be the one to guide me on what to do. Yes, I think I'd consult him right away. (OP003)<br>• Well, we try—if necessary, the doctor… I don't know, maybe he'll run some tests. I always follow my primary care doctor's advice first. He might say: "I don't know, let's do some tests, repeat the vitamin D, we'll see." Or he might say: "Go see the orthopedist." (OP004)<br>• My family doctor—with whom I have a good relationship—so I feel free to discuss a lot of things. (OP005)<br>• I trust the doctors… I rely on them and hope their advice is good. (OP008)<br>• Being able to speak openly with a doctor and have them say, "Look, this…" That's what I want—for example, like I told you… (OP011)<br>• She follows me fairly well… Anyway, my main point of reference is always my doctor. (OP013)<br>• Well, I would immediately call a specialist… an endocrinologist… I prefer a professional. (OP014)<br>• I immediately wrote to the doctor and said, "Unfortunately, I'm slipping backward a bit," and he tried to reassure me. (OP015)<br>• He told me, "Don't worry. Restart the treatment for a year, and repeat the scan in 18 months." I've never had any side effects from it. (OP015)<br>• I entrusted myself to the specialist—this professor who follows me—and I have to say, he's excellent. (OP016)<br>• Also my general practitioner, and the osteoporosis clinic at Tor Vergata. I spoke with the healthcare staff there—I discussed things and gladly followed their advice. (OP017)<br>• They're doctors—I have to rely on them. I have to trust what they say. I've always relied on my doctors. (OP018)<br>• And my doctor said, "Look, knowing your lifestyle and the way you do things, I wouldn't recommend it—because it might be a case of overmedication." And I (laughs) followed that advice. (OP020)

Volition	• I start with good intentions. (OP001)<br>• I know what the problem is, and as a result, I handle it myself. (OP002)<br>• All these things help the body cope better—if we want to call it a disease. (OP003)<br>• It's nothing special, really— in life, you can do anything, as long as you take proper care, right? (OP004)<br>• I'll try to keep myself well, to take care of myself… Yes, taking care of ourselves— I think it's about not neglecting ourselves. (OP005)<br>• But what should I do? I'll do it… At least for me, I really want to. (pause) I want to do it. (OP006)<br>• I'm doing what I can to try to improve this condition… I can't do more than that. (OP008)<br>• I'm doing this mostly because I know it could lead to problems— fractures, and so on. (OP009)<br>• If I don't do it, who's going to do it? (laughs) I have to try to take care of myself as best I can. (OP010)<br>• Thank you, and now, let's hope for the best. I'll try something... (sighs) we'll see. I'll try. I really will. (OP011)<br>• Personal needs—to feel self-fulfilled. That's the foundation of being human: if you feel fulfilled and satisfied, you're able to face many other situations around you. (OP014)<br>• It's a commitment the person takes on when dealing with illness. If a person can commit to their care, that's another form of support, another motivation—not necessarily to solve it, but to live well. (OP016)
Adaptation, Positive	• The only thing I've given up is the motorbike… So I gave up the bike—completely (OP001)<br>• I'm actually happy about it, I pay attention to these things…It doesn't weigh on me because I'm aware of what could happen, so it doesn't bother me at all (OP002)<br>• I'm quite meticulous when it comes to taking care of myself… Are they easy for you? Yes, yes, yes, yes (OP003)<br>• Well, you have to do the treatment, take the medication and be careful… it's nothing big. In life you can do everything, as long as you're cautious, right? (OP004)<br>• I don't really see it as an illness… I'll still try to take care of myself (OP005)<br>• I care about myself, that seems to cover it a bit. Let's say osteoporosis is just one part of taking care of myself (OP006)<br>• I just keep what I've got and, like I said, I take it philosophically. That's all (OP007)<br>• But then, during the day, if I keep moving, everything is fine (OP008)<br>• To have a better life—for myself and for those around me (OP009)<br>• These are positive aspects… I'm more careful now when I walk, where I place my feet. Before, I was always tripping (OP010)<br>• I've been trying to do something for a while now—mostly trying not to worsen the condition (sighs), to avoid it getting worse (OP011)<br>• And I also think that with osteoporosis things should be worse, but thank God, things are getting back to normal (OP012)<br>• I don't make a drama out of it, just like I haven't made a drama out of anything else that's happened (OP013)<br>• Look, I have my own philosophy: if I have a problem, I don't overthink it… But I've managed it well (OP014)<br>• I rushed to get treatment because the idea of a spinal fracture was unacceptable to me (OP015)<br>• The young people I work with on outside projects need someone who's always present, and I like taking care of myself in that way… I even quit smoking in the meantime, because unfortunately I had that bad habit (OP016)<br>• My first approach to osteoporosis was simply to accept that there was a problem… All in all, it's something I manage and it doesn't really cause me many issues (OP017)

	• If it doesn't show up, then I feel fine now… I feel good, no symptoms (OP018)<br>• I'm really the type of person who doesn't stress out—I always try to overcome things (OP019)<br>• I just get up half an hour earlier in the morning—simple as that. I like sleeping in, but I tell myself, "Oh! I have to take the medication." And then I find myself happily doing housework, ironing, getting things done, and the half hour flies by (OP020)
Networking for seeking information	• I'm trying to look into it a bit more to understand. We'll see (OP001)<br>• I study—I really do (laughs). I read a lot and stay informed (OP005)<br>• Just from hearing a few things here and there, I realized there are centers… of course I try to find the reasons for this (OP011)<br>• I read that, and now I think that when I have these, let's say (sighs), these sensations of pain in my teeth, it might be caused by that—otherwise I wouldn't have known (OP012)<br>• But according to what I've read, these things are prescribed (OP013)<br>• My knowledge about osteoporosis has led me to understand that, naturally, it progresses with age (OP014)<br>• So, I go to the general practitioner (sighs)... I'm telling you... again, I check myself regularly—I read. Like, the mammogram and everything I'm supposed to do, I always do the bare minimum they say we should do; but I don't go overboard either (OP015)<br>• I've really looked into it—partly because I enjoy it too (OP016)
Clinical guidance	• All the doctors (laughs), all of them including the pharmacist (laughs)... today to ask him for advice on this medicine (OP001)<br>• So basically it was advice from my general practitioner and the cardiologist (OP003)<br>• Then if there are problems, he says "come in for a check-up", you know (OP004)<br>• Everything is connected, thyroid, heart, osteoporosis—it's all linked. Both my GP and my cardiologist tell me that (OP004)<br>• They said that this year they found some improvements, so I just follow what they said (OP008)<br>• I try to do what they tell me… I do what they tell me to do (OP009)<br>• "Well, this is really bad," he said, "At least did the doctor give you the medicine?" I said, "Actually, no," and then he had me repeat the bone density scan and gave me alendronic acid, which I'm taking (OP010)<br>• Doctors also told me: the bone gets stimulated… more things, according to what doctors say (OP011)<br>• They told me I have osteoporosis, they told me to be careful when… when I walk, to be careful not to fall, because… (OP012) Always consult the experts (OP013)<br>• …as soon as I saw the bone density scan, I immediately wrote to the doctor and said "unfortunately I'm going a bit backwards," he reassured me a little (OP015)<br>• Primary care doctors must take care to inform women (OP016)<br>• I did it after a year and already from the first scan you could see signs… I followed that advice gladly (OP017)<br>• They tell me "Keep going like this," if things are going well. I ask how things are going, they say "good," they say "good, keep going like this"… if that's right (OP018)

Category: Osteoporosis treatment	
Effective remedies	• A new drug that will surely be effective… I take care of myself, except for that (OP001)<br>• But let's say the pharmacological therapy I'm doing seems to be working (OP003)<br>• I've seen a lot of improvement… and swimming helps too (OP004)<br>• I find it relaxing and it recharges me both psychologically and physically (OP006)<br>• I'll get better by taking these pills, that's all I hope (OP007)<br>• The positive aspects are exactly that I don't get fractures (laughs), that I don't get worse (OP008)<br>• But it's stable, in fact they haven't even changed my treatment… just monitoring it with the scan and the treatment I do (OP009) It says that it's a drug that is supposed to work, but of course it's not going to rebuild the bones. Still, it helps me get along a bit better, right? (OP010)<br>• If I stay still, if I stay warm, I seem to avoid the pain, it seems to go away. Maybe it's a placebo effect, I don't know, but still (OP011) Since I've started this last injection every six months… And as I already told you, I also take a basic supplement… things are going a bit better (OP012)<br>• The only thing that helps me is a corticosteroid injection, but we also have to be careful with that because it seems corticosteroids worsen osteoporosis. For pain, if I want to fight the pain, when there's strong inflammation, the only thing that helps me is corticosteroids… I think I can evaluate the source of the criticism (OP013)<br>• So, I always stick to that data, and I started doing some tests… they prescribed me denosumab (OP014)<br>• Having the chance to stop a worsening… Yes, I think I'm managing it because I'm monitoring myself (OP015)<br>• I've found great relief especially regarding the functional limitations that the pain caused me, sometimes I couldn't sleep at night because of the pain… with this therapeutic help I feel much, much better (OP016)<br>• Eating healthy foods, things that can help maintain my calcium levels (OP017)<br>• With the medicines they give me, I try to get along… I take pills once a week and then I take vitamin D (OP019)<br>• Now I've been taking alendronic acid with cholecalciferol for a year and the bone density scan has slightly improved (OP020)
Safety	• They suspended it and gave me, well, for that year they gave me some tablets (OP008)<br>• So far I haven't had any problems, fortunately. The doctor also told me: "Be aware that it could cause discomfort." But no, it went well (OP010)<br>• The second time I had the denosumab injection, it caused high blood pressure, it raised my blood pressure values (OP014)<br>• Clodronate… monthly vitamin D, 50 mg, and then walking every day (OP015)<br>• You know very well what happens with prolonged drug use not just on the gastric mucosa, but also on the intestines, so this combination of gastroprotectors and probiotics really helped me manage the symptoms better (OP016)<br>• Honestly, sometimes I take gastric protectors, if I have them, and other times I try to eat something to see if it goes away (OP017)<br>• Being careful not to do anything… I mean, dangerous (OP019)
Low out-of-pocket costs	• I don't know if it's really true that things have changed… so, I was entitled, let's say, but I paid the co-pay (OP015)<br>• The treatments are accessible, so… (OP017)

Self-administer	• Only denosumab every six months and then a base treatment every 15 days and once a month (OP002)<br>• I take it in soluble sachets in water and that bothered me, so we opted for tablets, the chewable kind… we found them… yes, they're chewable tablets (OP004)<br>• Because they are medications I can take (OP008)<br>• The only thing that helps me is a corticosteroid injection, but we also need to be careful there because it seems that corticosteroids worsen osteoporosis. For the pain, if I want to fight the pain when there's strong inflammation, the only thing that helps is corticosteroids (OP013)<br>• Besides denosumab, I started therapy and fortunately they found that after two injections over six months… I have both vitamin D and calcium within normal range… I have a fairly sharp mind because I always study (OP014)<br>• Clodronate… vitamin D monthly 50 mg and then walking every day (OP015)<br>• Also having to take medication… gastroprotectors, but also very important dietary supplements (OP016)<br>• Honestly, sometimes I take stomach protectors if I have them, and other times I try to eat something to see if it passes (OP017)<br>• I get an injection every six months and a tablet for 20 days, one a day. Ah… and also cholecalciferol (OP018)<br>• I take tablets once a week and then I take vitamin D… otherwise I take some anti-inflammatories that help me a little (OP019)<br>• I take something once a week, alendronic acid (OP020)
Strategies to facilitate adherence	• In the morning, when I have to take alendronate, I don't place the other medications there, so when I don't find the boxes on the nightstand, I remember. Plus, I leave the alendronate box and an empty glass next to the sink in the bathroom, because the first thing I do is brush my teeth, to remind myself not to have breakfast. Otherwise, I'd eat and forget the alendronate. So I use these memory tricks (OP001)<br>• I remember I have to take them and so I do it accordingly (OP002)<br>• It has become a routine (OP003)<br>• Well, nothing really, because it's not such a burdensome therapy. I take the calcium/vitamin D tablet mid-morning, and once a week I take my 25 drops of clomiphene (OP003)<br>• I spread it on bread, it doesn't bother me. Many people find it unpleasant, but not me. I think it's subjective (OP004)<br>• I walk with my phone so I don't think about anything (laughs), that way everything passes and I feel nothing (OP007)<br>• I have a planner where I write down every 15 days at 3:30 PM to take cholecalciferol (OP008)<br>• Yes, yes, I put it in the kitchen near the coffee machine (laughs), so I can't forget (OP010)<br>• The only thing that helps me is a corticosteroid injection, but we have to be careful there too, because it seems corticosteroids worsen osteoporosis. For pain, when there's a strong inflammation, the only thing that helps me is corticosteroids (OP013)<br>• So I always follow that data and started doing some further tests (OP014)<br>• I have quite a sharp mind because I'm always studying (OP014)<br>• I'm quite disciplined, so in the end, I do those check-ups anyway (OP015)
**Category: Exercise**	
Adequate support systems	• I go to the senior center, you know... the usual one where they do a bit of everything, especially working on the joints, and there's a lot of walking, while they tell you "do this, do that"... many, many, so many... No day is like the other (OP018)

Adequate network resources	• Well, I would immediately call a professional... the endocrinologists... (OP014)<br>• I mean, I go to the general practitioner (sigh)... I'm telling you... I don't... I repeat. I monitor myself in general... I read, I mean mammograms and what needs to be done, I always do the minimum of what they tell us to do; but I don't overdo it (OP015)<br>• I go to the senior center, you know... the usual one where they do a bit of everything, especially working on the joints, and there's a lot of walking, while they tell you "do this, do that"... many, many, so many... No day is like the other (OP018)
Positive emotions regarding physical activity	• These are all things that, in my opinion, help the body cope better, if we want to call it a disease (OP003)<br>• Lots of physical activity, in fact I enrolled in a swimming pool. I've always gone, but now I go regularly (OP005)<br>• I don't make a big deal out of it, just like I've never made a big deal out of anything else, of any other event (OP013)<br>• I don't do big jumps, but I can do little ones. So, it's also about adjusting your abilities, but absolutely not neglecting them. That's kind of my philosophy (OP014)<br>• We enjoy this, especially walking, running, walking along the beach, a bit of sun, because that too is essential to our therapy... I used to be more anxious (sigh), but now everything feels much calmer for me (OP016)<br>• Physical activity, fortunately, is something that suits me very well, so among the things I need to be careful about, this one weighs on me the least... I generally love any kind of activity (OP017)<br>• It shows up from time to time, otherwise I feel just fine now... I feel well, it doesn't bother me at all (OP018)<br>• I do physical activity, meaning I do yoga and walk a lot. I walk, I also do Nordic Walking, I like it. I'm not particularly sedentary without being a fitness fanatic either (OP020)
Positive reactions regarding physical activity	• With the therapy I did, and the physical exercise I followed (OP004)<br>• Having recently lost my husband, and still having children to think about, I don't want to become a burden on them. That's also a trigger for maintaining physical health... So that's a big motivation for me, right? (OP005)<br>• I find it to be something important (OP013)<br>• I dedicate time to myself. When I feel like the whole world is collapsing on me — and that may sound like a metaphor — I create my own spaces. My spaces are my home, my world, what I have put into my home, the colors I've chosen for it, and that makes me happy (OP014)<br>• I also quit smoking in the meantime, because unfortunately I had that bad habit in my life (OP016)<br>• My first approach to osteoporosis was really about entering a mindset of awareness, acknowledging that there was a problem... All things considered, it's something I manage, and it really doesn't cause me many problems (OP017)<br>• I don't do anything because I don't need anything, and nothing is required of me. I don't notice anything. I can say that after these procedures, I feel great, and my legs feel like they're twenty again... No, for me everything's the same. I don't feel any discomfort at all (OP018)<br>• I'm just the kind of person who doesn't let things get to me — I always try to overcome everything (OP019)<br>• I'm not particularly sedentary, though I'm not a fitness fanatic either (OP020)
Customized exercises	• Yes, the physiotherapist. I went to the orthopedist, and he recommended the physiotherapist more, the suggestions on how to do the exercises... anyway, the doctor and the physiotherapist guide you through the process (OP004)<br>• I also did a water exercise program, hydro-kinesis (OP004)<br>• You learn the proper exercises for this kind of condition (OP005)<br>• I put strain on my spine, so I chose swimming (OP008)

	• Now I try to prevent it as they told me, by walking, moving, doing some physical exercise and so on (OP011)<br>• Walking, because I can't play sports, but I can walk a lot (OP013)<br>• Moving around, walking (OP013)<br>• I go hiking in the mountains (OP014)<br>• I ride my bike (OP014)<br>• I swim and dance (OP014)<br>• I dance (OP014)<br>• I even spend a couple of hours jumping around (OP014)<br>• I take vitamin D monthly (50 mg) and walk every day (OP015)<br>• Now that my routine has changed, let's say... but during the day, I always try to get my 45 minutes in (OP015)<br>• I try to stay active anyway (OP015)<br>• Also, by going dancing a bit, doing some pilates, he adjusted my posture a bit, so I try to keep myself a little more upright (OP015)<br>• So, diet and exercise — the management of my condition basically involves this: the healthiest lifestyle possible and outdoor physical activity (OP016)<br>• Taking long walks, everything that helps keep the bones healthy (OP017)<br>• Physical activity, luckily, is something that really suits me, so among the things I need to pay attention to, this one is the least burdensome (OP017)<br>• I take long walks and that helps me a lot, also psychologically (OP017)<br>• When I can, I also do posture training, I do posture training (OP017)<br>• With sports, with movement (OP017)<br>• I also go to the gym twice a week (OP018)<br>• I go to the senior center — they do a bit of everything there, especially working on the joints, with lots and lots of walking, while they guide you: “do this, do that”... so many activities... no day is the same (OP018)<br>• I do physical activity, meaning I do yoga and walk a lot. I walk, I also do Nordic Walking, I like it. I'm not particularly sedentary, but not a fitness fanatic either (OP020)
Encouragement in physical activity	• They told me that walking is good for me (OP003)<br>• Yes, the physiotherapist. I went to the orthopedist, and the orthopedist recommended the physiotherapist more, the guidance on how to do the exercises... instead, he said “don't be afraid” and “do it,” he said “if you feel pain, that's another matter” (OP004)<br>• Walking, because they told me that by walking I stimulate them, and I used to do physical activity before too (OP011)<br>• When I saw that bone density scan, I immediately wrote to the doctor and said “unfortunately I'm going a bit backwards,” and he consoled me a bit (OP015)<br>• He told me, “don’t worry. Resume the treatment for a year and do another scan in 18 months” (OP015)<br>• I go to the senior center — the usual one where they do a bit of everything, especially working on the joints, and there’s a lot of walking. They say “do this, do that”... so many activities. No day is the same (OP018)<br>• As my father used to say, “Keep going,” so we hope to carry on with the awareness that I’m no longer young... but let’s hope to have the physical and mental strength to face life in the best way possible (OP020)

Guidance from physical therapists	• Yes, the physiotherapist. I went to both the orthopedist and the orthopedist recommended more the physiotherapist, the advice on how to do the exercises... in any case, the doctor and the physiotherapist guide you through the path. They also tell you... like, yes, it's good for you (OP004)<br>• Walking, because they told me that by walking I stimulate them, and I used to do physical activity before too (OP011)<br>• Then, by going to dance a bit, doing some pilates, he straightened me up a bit, and now I tend to keep a better posture (OP015)<br>• I go to the senior center — the usual one where they do a bit of everything, especially working on the joints, and there's a lot of walking. They tell you "do this, do that"... so many activities. No day is the same (OP018)
Mindful exercise	• Yes, this I can, I can perceive it. I repeat, I'm careful because it's always been... I mean, it's a kind of attention I've always had... I can perceive the changes... I trust and rely a lot on my own feelings (OP005)<br>• By doing yoga, yoga really makes you... it makes you aware because certain things you can only do by maintaining a certain stillness (OP006)<br>• I practiced meditation and yoga for many years…Well, as I said, yoga — so I also learned how to move when bending, lifting weights, walking, and breathing (OP014)<br>• When I can, I also do postural gymnastics, I do postural gymnastics (OP017)<br>• I do physical activity, meaning I practice yoga and walk a lot. I walk, I also do Nordic walking. I like it. I'm not particularly sedentary without being a fitness fanatic either (OP020)
**Category: Confidence in one's ability**	
Adequate consumption of milk and dairy products	• What I really can't give up is milk (OP003)<br>• Oat milk, almond milk. And also cheese (OP004)<br>• Breakfast based on skimmed milk (OP013)<br>• Choosing foods that contain calcium and therefore being careful, very careful with my diet… foods I had introduced, or in any case, yogurt, dairy products (OP017)
Adequate exposure to sunlight	• Going out to get some sun (OP004)<br>• The only thing I can do is stay in the sun as long as possible (OP013)<br>• Honestly, sun for life (laughs), because it's a source of well-being, it's good for the mind, the spirit, and the bones. That's important. As soon as I have two minutes, even on the balcony, I go out and sit in the sun. That's something really essential, truly indispensable…I used to sunbathe (OP014)<br>• We enjoy it, especially running, walking on the beach, some sun, because that too is fundamental in our therapy… we can't afford anything fancy, but walks outdoors, a short run, sun exposure (OP016)<br>• Staying in the sun, sun exposure for vitamin D, for support… with sun, walks by the sea (OP017)
Nutritional counseling	• It was the cardiologist, because he told me that gaining too much weight, yes, yes… anyway, eating helps me physically to better handle my body (OP003)<br>• The doctor recommended cheese and Parmigiano Reggiano (OP004)<br>• As I was told, I eat cheese (OP009)<br>• When you're a child: "Drink milk to strengthen your bones," right? So I thought it was something like that (OP010)<br>• Following a balanced diet (OP016)

	• Choosing foods that contain calcium and paying close attention to nutrition... they also gave me some other suggestions for supplementation (OP017)<br>• As for diet, I've been to the dietitian several times because I'm slightly overweight. But I try to eat well, I try to avoid sweets, even though I love them (OP020)
**Category: Self-management strategies**	
Faith healing to relief pain	• But I don't have any issues, thank God, I don't suffer (OP019)
Osteoporosis prevention	• I think a more targeted campaign about this disease should be launched, because I believe it would benefit the health system more if people take care of themselves, rather than ending up needing disability benefits, hospital treatments, or therapies (OP004)<br>• If I had started treatment earlier, I probably wouldn't have ended up in this situation. Back then they didn't monitor vitamin D levels unless it was specifically requested. Now I can see things have changed (OP005)<br>• What I mean is that my current approach is more preventive than curative (OP006)<br>• At least it was caught in time, I hope. I want to stay better, not get worse (OP007)<br>• "I always say: please, go to the doctor, ask for something for your bones, because it's not something pleasant." Yes, I say it to all my friends: "Please, get a bone scan done, because…" Yes, I say it (OP010)<br>• I've been trying to do something for a long time. Mainly to avoid making things worse… to prevent further damage. I should've gone to a specialized center right away if someone had told me back then (OP011)<br>• From what I've read, these treatments are prescribed (OP013)<br>• I repeated the bone scan and saw that my values had decreased (OP014)<br>• Luckily, the diagnosis came early… I still go for screening. I mean, I get a bone scan every six months (OP016)<br>• I had it done about a year later and already from the first bone scan there were signs (OP017)<br>• The condition hasn't worsened, it's stayed stable, like it was 5, 6, even 7 years ago when the osteoporosis began. My levels have always stayed the same… Of course, I need to keep getting check-ups and take the medications they prescribe, because vitamin D helps, and that little tablet—maybe it's calcium or whatever—helps too. And I have to be careful not to do anything dangerous (OP019)<br>• That's why it's one of the check-ups I do preventively… always as a prevention. I go for osteoporosis screenings just like I do for breast cancer (OP020
Trying not to think about illness	• You shouldn't become obsessive in that sense, I mean, constantly thinking about your illness, whatever it may be (OP005)<br>• Look, I have my own philosophy: if I have a problem, I don't dwell on it too much… I try not to focus my thoughts constantly on the pain (OP014)
